# Molecular sensors reveal the mechano-chemical response of *Phytophthora infestans* walls and membranes to mechanical and chemical stress

**DOI:** 10.1016/j.tcsw.2021.100071

**Published:** 2022-01-07

**Authors:** Lucile Michels, Jochem Bronkhorst, Michiel Kasteel, Djanick de Jong, Bauke Albada, Tijs Ketelaar, Francine Govers, Joris Sprakel

**Affiliations:** a Physical Chemistry and Soft Matter, Wageningen University & Research, Stippeneng 4, 6708 WE, Wageningen, the Netherlands; b Laboratory of Phytopathology, Wageningen University & Research, Droevendaalsesteeg 1, 6708 PB, Wageningen, the Netherlands; c Laboratory of Cell Biology, Wageningen University & Research, Droevendaalsesteeg 1, 6708 PB, Wageningen, the Netherlands; d Laboratory of Organic Chemistry, Wageningen University & Research, Stippeneng 4, 6708 WE, Wageningen, the Netherlands

**Keywords:** Cell wall, Plasma membrane, Fluorescence Lifetime Imaging, Crop protection agents, Mechanobiology, Molecular mechanosensors, *Phytophthora infestans*, Plant pathogens

## Abstract

*Phytophthora infestans*, causal agent of late blight in potato and tomato, remains challenging to control. Unravelling its biomechanics of host invasion, and its response to mechanical and chemical stress, could provide new handles to combat this devastating pathogen. Here we introduce two fluorescent molecular sensors, CWP-BDP and NR12S, that reveal the micromechanical response of the cell wall-plasma membrane continuum in *P. infestans* during invasive growth and upon chemical treatment. When visualized by live-cell imaging, CWP-BDP reports changes in cell wall (CW) porosity while NR12S reports variations in chemical polarity and lipid order in the plasma membrane (PM). During invasive growth, mechanical interactions between the pathogen and a surface reveal clear and localized changes in the structure of the CW. Moreover, the molecular sensors can reveal the effect of chemical treatment to CW and/or PM, thereby revealing the site-of-action of crop protection agents. This mechano-chemical imaging strategy resolves, non-invasively and with high spatio-temporal resolution, how the CW-PM continuum adapts and responds to abiotic stress, and provides information on the dynamics and location of cellular stress responses for which, to date, no other methods are available.

## 1 Introduction

Host infection by biotrophic filamentous plant pathogens invariably commences with host entry, a complex process in which the pathogen utilizes mechanical weaponry to breach the protective barrier posed by the plant surface, guided and aided by biochemical interactions between pathogen and host. Understanding the nature and mechanisms of host invasion is an important route to find new solutions to control plant pathogens and mitigate yield losses resulting from plant diseases. From this vantage point, obtaining a complete picture of the plant–microbe interactions during host entry requires study of the invasion process from the perspective of the host and that of the pathogen, and taking both mechanical and chemical interactions into account.

Among the most damaging plant pathogens is the oomycete *Phytophthora infestans*, causal agent of late blight in potato and tomato, and responsible for large yield losses and economic damage (Haverkort et al., 2009). Despite tremendous progress in unravelling its interaction with host plants and continued efforts to breed resistant crops, *P*. *infestans* remains challenging to control, in part due to its large and rapid genetic adaptability (Haas et al., 2009). While there are many studies on the invasion mechanisms of *Phytophthora* spp from a genetic, biochemical and cell biological perspective, very little is known about the strategies they exploit to gain mechanical entry into plants.

Various molecular-genetic and biochemical approaches as well as omics analyses have been widely used to identify, on the one hand, *Phytophthora* genes encoding effectors and other proteins required for pathogenicity (Judelson et al., 2008, Haas et al., 2009, Resjö et al., 2017, Wang and Wang, 2018, McGowan and Fitzpatrick, 2020) and, on the other hand, plant genes implicated in immunity and susceptibility to late blight (Vleeshouwers et al., 2011, Sun et al., 2016, Witek et al., 2016). Additionally, the molecular mechanisms underlying the manipulation of plant immunity by effectors have been widely studied (Du et al., 2015, Boevink et al., 2020, Fabro, 2021, Petre et al., 2021). Studies on genetics, secreted effectors and cytoskeletal regulation are vital to elucidate essential components of the invasion machinery of *P. infestans*. Yet, connecting how these components work in synchrony during infection, and how they couple to the mechanical process necessary to breach the plant surface remains challenging.

In a recent study (Bronkhorst et al., 2021), we took a first step to unravel the mechanics of host entry by *Phytophthora* spp. Using a combination of surface deformation imaging, molecular fracture sensors and modelling, we uncovered a mechanism, coined as ‘naifu’-mechanism, that *Phytophthora* spp. use to enter their hosts. This mechanism relies on an oblique application of pressure to the surface, at a distinct off-perpendicular angle, that facilitates host entry by reducing the invasive pressure required to break the host surface. The discovery of this mechanism gives rise to a plethora of new questions on how *Phytophthora* perceives and processes mechanical signals at the pathogen-host interface into intracellular mechano-biological responses.

Resolving this challenge requires new tools that provide insights into local mechano-chemical properties at the surface of and inside the pathogens during mechanical invasion, ideally suitable for live-cell imaging and with high spatio-temporal resolution. In a previous study (Michels et al., 2020), we introduced a toolbox of four molecular mechanosensors that can resolve complex micromechanical patterns in plant cells during a range of cellular processes. The photoluminescence properties of such sensors, and in particular their fluorescence lifetime, vary according to the local free volume available around the molecules. For that reason, these sensors have been used to measure e.g. porosity or crowding density within cellular compartments in plants. These approaches remain to be utilized for the study of pathogens during invasive growth.

In addition to the understanding of their invasion mechanics, the identification of novel chemical control agents is an important avenue to combat *Phytophthora* pathogens more effectively (Grünwald et al., 2007, Wang et al., 2014). Suitable control agents typically act on specific parts of the pathogen anatomy. Usually, their efficacy is studied by monitoring alterations in colony propagation and cell morphology, as well as in infection assays in green houses and field trials. However, these approaches do not resolve the site- and mode-of-action of the compounds. Moreover, fast screening at limited cost remains challenging and hinders deep screening of chemical control agents. In that regard, mapping spatio-temporal variations within cellular compartments using molecular mechanosensors could reveal the outlines of the pathogen’s mechano-chemical reaction to treatment relatively quickly and inexpensively, and provide a completely novel aspect to evaluate drug efficacy.

In this paper, we introduce a pair of two synthetic molecular sensors that are capable of resolving the intracellular mechano-chemical response of the cell wall (CW) – plasma membrane (PM) continuum in *P. infestans* during hyphal and invasive growth and in response to various chemical treatments. The first one is a cell-wall binding molecular rotor (Michels et al., 2020) that reports on spatial and temporal changes in CW porosity and composition while the second one is a solvatochromic PM probe that reveals changes in membrane chemical polarity and molecular order.

**Fig. 1:**
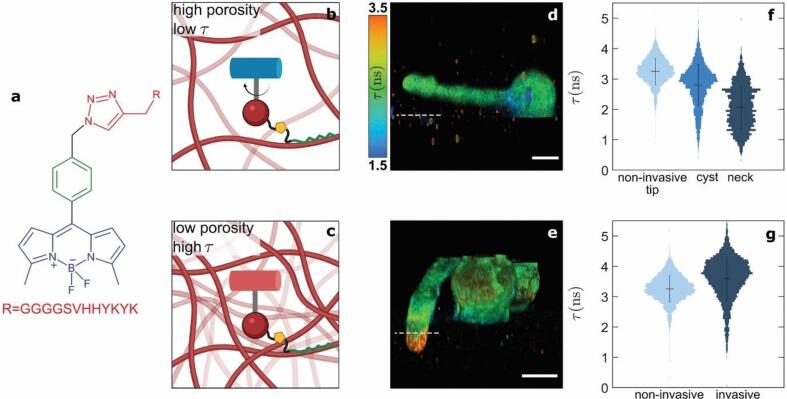
Mapping of spatial variations in the cell wall mesh size of *Phytophthora infestans* germlings using the molecular rotor CWP-BDP. (a) Chemical structure of CWP-BDP. (b,c) Schematic illustrations showing the molecular mechanism by which CWP-BDP reports mesh sizes. 3D fluorescence lifetime mesh size map of *Phytophthora infestans* germlings (d) upon growth at the PDMS substrate surface and (e) upon invasion. Scale bars = 5 µm. The colorscale translates the fluorescence lifetime values expressed in ns. The white dashed line is used to indicate substrate surface location (f) Fluorescence lifetime probability distributions obtained in different regions of the germlings (N = 30 cells). (g) Fluorescence lifetime probability distributions obtained in the tip of germ tubes before and after invasion (N = 30 cells).

The CW molecular sensor CWP-BDP is a phenyl-substituted borondipyrromethene (Ph-BODIPY) molecular rotor, substituted on the phenyl ring by a peptide mimicking the pectin binding domain of extensins, a family of glycoproteins that are highly abundant in plant CWs (MacDougall et al., 2001). As such, it has been designed to target and bind specifically to the wall of plant cells. The molecular rotor, whose mode-of-action is described in detail elsewhere (Michels et al., 2020), offers a mechano-optical coupling that allows qualitative measurements of local CW mesh sizes, using a fluorescence lifetime read-out (Fig. 1a–c).

**Fig. 2:**
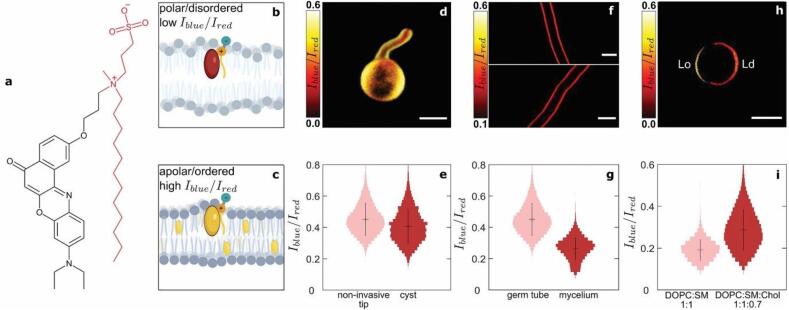
Mapping of spatial variations in the plasma membrane chemical polarity of *Phytophthora infestans* germlings using the solvatochromic probe NR12S. (a) Chemical structure of NR12S. (b,c) Schematic illustrations showing the mechanism by which NR12S reports changes in chemical polarity and lipid phase. (d) 3D intensity ratio chemical polarity map of *Phytophthora infestans* germlings upon growth at the PDMS substrate surface. Scale bar = 5 µm. The colorscale translates the intensity ratio values. (e) Intensity ratio probability distributions obtained in different regions of the germlings (N = 30 cells). (f) Intensity ratio chemical polarity map of *Phytophthora infestans* mycelium. Scale bars = 10 µm. (g) Fluorescence lifetime probability distributions obtained in the young germ tubes tip (N = 30 cells) versus mycelial cells grown on agar (3 mycelia, N = 30 images). (h) Intensity ratio chemical polarity map of DOPC:SM:Cholesterol = 1:1:0.7 (molar ratio) synthetic vesicles exhibiting phase separation between a cholesterol-rich ordered (Lo), and a cholesterol-poor disordered (Ld) phases. Scale bar = 5 µm. (i) Intensity ratio probability distributions obtained in DOPC:SM = 1:1 (molar ratio) (N = 20 vesicles) versus DOPC:SM:Cholesterol = 1:1:0.7 (molar ratio) (N = 20 vesicles) synthetic vesicles.

The PM molecular sensor NR12S is a solvatochromic Nile Red-based probe developed by Kucherak et al. (Kucherak et al., 2010) (Fig. 2a). In its design, the Nile Red unit is decorated with a long alkyl tail and a zwitterionic group, which allows for specific staining of the PM and restricts flip-flopping of the dye from the outer to the inner leaflet, thereby reducing subsequent incorporation in intracellular membranes. This probe exhibits a shift in the wavelength of maximum emission in response to changes in the local chemical polarity of its surroundings. The read-out for this probe consists of ratiometric imaging, in which the total emission of the dye is split into two channels. Changes in membrane chemical composition and lipid phase both impact the chemical polarity of the probe microenvironment, triggering a change in the intensity ratio between the blue and red channels (Fig. 2b & c). Kucherak et al. (Kucherak et al., 2010) used these properties to image variations in PM lipid order and cholesterol content in mammalian cells. Here we extend the use of this probe to walled cells.

By using a combination of both molecular sensors we show how invasive growth of *P. infestans* generates a localized sub-cellular response at the pathogen-host contact point as a gateway for mechanical signal internalization, and how certain chemicals elicit specific responses in the CW-PM continuum. These new tools can contribute to bridging the gap between the genetic, biochemical, cell biological and mechanical aspects of host entry by devastating plant pathogens.

## 2 Materials and methods

### 2.1 Synthesis of fluorescent molecular sensors

The CW rotor, CWP-BDP, was synthetized as reported in Michels et al. (Michels et al., 2020), and the PM ratiometric sensor, NR12S, was synthesized following the protocol reported by Kucherak et al. (Kucherak et al., 2010). A comprehensive overview of all experimental details, including synthesis and chemical characterization of the molecular sensors are provided in Methods **S1** and **S2**, and Figures S1-18. Absorbance and emission spectra of the sensors are given in Figure S19.

### 2.2 *P. infestans* culture and cyst generation

*P. infestans* wild-type strain 88,069 (*P. infestans-wt*), was maintained on rye sucrose agar (RSA) medium (Caten and Jinks, 1968) supplemented with Vancomycin (20 µg ml^−1^), Ampicillin (100 µg ml^−1^), and Amphotericin A (10 µg ml^−1^). Cultures were grown for 10 days at 18 °C in the dark, and maintained by regularly transferring a 5x5 mm mycelial plug to new plates.

Zoosporogenesis was initiated by adding sterile tap water (4 °C) (10 ml for 100-mm-diameter plates, 5 ml for 60-mm-diameter plates) to a 10 to 14-day-old culture. To stimulate zoospore release the culture was incubated at 4 °C for 3 h, whereafter the zoospore suspension was collected in a 50 ml Falcon tube. Zoospore encystment was triggered by manually shaking the tube for 1 min. The suspension was diluted 5x with milli-Q water, to reach a concentration of about1 × 10^5^ cysts ml^−1^ for experimental use.

In parallel, for mycelium PM imaging with NR12S, *P. infestans-wt* mycelium was grown while exposed to a relatively low concentration of β-sitosterol. This was done by placing two mycelium plugs (about 2 mm diameter) from a 7 day old *P. infestans* culture grown on Plich medium (Van der Lee et al., 1997) lacking sterols on opposite sites of a disk (1 cm diameter, 0.5 cm thickness) of Plich agar medium supplemented with 0.2 µg ml^−1^ β-sitosterol (from a stock of 2 g l^−1^ β-sitosterol in DMSO) and allowing hyphae to grow into the disk. The final percentage of DMSO in the medium was 0.1% (v/v). Disks were incubated at 18 °C in the dark for three days before staining with NR12S. Plich medium is transparent, as opposed to RSA medium, which allowed for imaging without signal contamination from the agar substrate.

### 2.3 Substrate preparation

All reactive PDMS polymers were purchased from Gelest Inc. and used as received.

For observation of invasive growth, artificial substrates that mimic the hydrophobicity and stiffness of plant leaves were used. Our previous work has shown that these substrates elicit a similar invasion response as real host surfaces (Bronkhorst et al., 2021). Stiff PDMS elastomer surfaces were prepared from a commercial 2-component polydimethyl siloxane rubber (Sylgard 184, Dow Corning) in a 10:1 base:crosslinker ratio. The two components were mixed by vortexing for 3 to 4 min. Bubbles and dust were removed from the viscous mixture by centrifugation at 1000**g** for 10 min. No.1 18x18 mm glass coverslips were cleaned before spincoating with the elastomer precursor mixture; coverslips were rinsed sequentially with isopropyl-alcohol (IPA), Milli-Q deionized water, and IPA, and subsequently dried in a nitrogen stream, followed by heating to 60 °C for 5 min. Coverslips were pre-treated using an O_2_/N_2_ plasma for 1 min, and then spincoated with 130 µl of mixture, at 500 rpm for 30 s followed by 2000 rpm for 2 min, to reach a 33 µm layer thickness (as determined by confocal microscopy). The Young’s modulus, representing the elastic constant for uniaxial tensile deformations, of these substrates was measured on a home-built indentation set-up, as described previously (Boots et al., 2019), and determined to be 1.5 MPa. The samples were placed inside a vacuum chamber for at least 30 min to remove air bubbles, before curing at 50 °C overnight.

For observations of non-invasive hyphae, solvent-free PDMS elastomer surfaces were prepared following the procedure reported by Cai et al. (Cai et al., 2015). For preparation of the precursor, the following components were used; Backbone: vinylmethylsiloxane–dimethylsiloxane copolymer, trimethylsiloxy terminated, c. 300 vinyl groups per molecule, Mw ≈ 50 000 g mol^−1^ (VDT-5035). Side chain: monohydride-terminated poly(dimethylsiloxane), Mw ≈ 4750 g mol^−1^ (MCR-H21). Crosslinking chain: hydride-terminated polydimethysiloxane, Mw ≈ 17 200 g mol^−1^ (DMS-H25). The PDMS linear polymers were mixed at a VDT-5035:MCR-H21:DMS-H25 = 1:13.3:1.72 mass ratio to achieve a 1:140:5 backbone:side chains:crosslinks number ratio, and a final Young’s modulus of 15 kPa. The three components were mixed by vortexing for 3 min. Bubbles were removed by sonicating the sample for 10 s. The Karstedt’s catalyst was added to the precursor mixture at a concentration of 5 µl g^−1^ from a 2% platinum solution in xylene. The solution was again thoroughly mixed and sonicated for 10 s. Plasma cleaned No.1 coverslips were then spincoated with 130 µl of mixture, at 500 rpm for 30 s, followed by 2000 rpm for 2 min. The samples were eventually cured at 70 °C for 48 h.

### 2.4 Cell incubation and fluorescence imaging

For imaging experiments, a 80 µl droplet of a cyst suspension (≈10^5^ cysts per ml) was deposited onto the elastomer surface, followed by placement inside bespoke 3D printed sample chambers (Fig. S20) to minimize water evaporation. The cysts were left to germinate for at least 1 h. For staining, a portion of the aqueous phase of the droplet, 50 µl, was replaced three times with a solution of either CWP-BDP or NR12S, dissolved at 10 µmol l^−1^ in water. The staining was performed for 15 min (CWP-BDP) or 7 min (NR12S), after which any unbound dye was removed by replacing 50 µl of the droplet four times with water.

To image PM polarity in *P. infestans* mycelium using NR12S, 80 µl of NR12S staining solution at 10 µmol l^−1^ in water were deposited onto the mycelium grown on a solid agar pad. Staining was performed for 7 min and the dye solution was subsequently removed. The agar pad was placed upside down on a coverslip for imaging.

Two-dimensional Fluorescence Lifetime Imaging (FLIM) of CWP-BDP stained samples was performed on a Leica TCS SP8 inverted confocal microscope coupled to a Becker&Hickl TCSPC lifetime module (SPC830). Samples were excited with a 514-nm pulsed laser source (pulse duration < 1 ps) with a repetition rate of 40 MHz, and fluorescence emission was captured through a 63 × water immersion objective (numerical aperture = 1.2). A line scanning speed of 400 Hz was used and the emission was collected, using a spectral window extending from 518 nm to 600 nm, onto a Leica HyD SMD hybrid photodetector. Acquisition time was fixed at 120 s for each 256 × 256 pixel image. FLIM images were processed using the SPCImage 7.1 software to fit the fluorescence decay curves in each pixel with a two-component exponential decay.

Three-dimensional FLIM imaging, using FastFlim, for CWP-BDP, as well as 2D- and 3D- ratiometric imaging with NR12S were performed on a Leica TCS SP8 Two-photon inverted confocal microscope. Samples were excited with a Chameleon Ti:Sapphire pulsed laser source (pulse duration = 140 fs), at either 810 nm (CWP-BDP) or 830 nm (NR12S), with a repetition rate of 80 MHz. Fluorescence was captured through a 40 × water immersion objective (numerical aperture = 1.2). A line scanning speed of 400 Hz was used and the emission was collected in a Leica 4 Tune detection unit equipped with Leica HyD SMD hybrid photodetectors. The emission of CWP-BDP was collected in a single photodetector, using a spectral window extending from 500 nm to 600 nm. The emission of NR12S was collected in two separate channels, using spectral windows extending from 500 nm to 585 nm, and from 585 nm to 700 nm respectively. The acquisition time was fixed at 15 s for each 256 × 256 pixel image. For three-dimensional image stacks, the z-step was set to 0.5 µm.

FLIM images obtained with CWP-BDP imaging were processed using the SP8 Falcon software that determines lifetimes based on the average arrival time of photons. Ratiometric images obtained with NR12S were constructed from the recorded intensity images using a custom Matlab routine that divides the photon count in each pixel of the 500–585 nm (so-called ‘blue’) channel image, by the photon count in the corresponding pixel of the 585–700 nm (so-called ‘red’) channel image. Resulting images are reported in a false-color scale that represents the mean CWP-BDP fluorescence lifetime (in nanoseconds), or the NR12S intensity ratio for each pixel.

Control images for the evaluation of autofluorescence intensities and fluorescence lifetimes were recorded on unstained specimens with the same imaging conditions and the highest laser intensity used, i.e. <10 µW (single-photon microscope) or 5 mW (multiphoton microscope) at the sample level. The autofluorescence levels in the CW and PM were too low to perturb our results (Fig. S21).

### 2.5 Giant Unilamellar vesicle (GUV) preparation

Sphingomyelin (SM) and 1,2-dioleoyl-*sn*-glycero-3-phosphocholine (DOPC) stock solutions in chloroform were purchased from Avanti Polar Lipids and used as such. Cholesterol was purchased from VWR. Giant Unilamellar Vesicles (GUV) were formulated via agarose gel swelling using the method of Horger et al. (Horger et al., 2009). Two types of vesicles were formulated, with molar ratios of DOPC:SM = 1:1 and DOPC:SM:Chol = 1:1:0.7, respectively. For the cholesterol-free solution, DOPC (1.2 mmol l^−1^, 57 mg of 25 g l^−1^ stock solution in chloroform) and SM (1.2 mmol l^−1^, 137 mg of 10 g l^−1^ stock solution in chloroform) were mixed in chloroform (370 μl). For the cholesterol-supplied solution, DOPC (1.0 mmol l^−1^, 48 mg of 25 g l^−1^ stock solution in chloroform), SM (1.0 mmol l^−1^, 117 mg of 10 g l^−1^ stock solution in chloroform), and cholesterol (0.7 mmol l^−1^, 42 mg of 10 g l^−1^ stock solution in chloroform) were mixed in chloroform (361 μl).

In parallel, Type IX-A ultralow melting agarose (gel point, Tg ≤ 20 °C; melting point, Tm ≤ 62 °C; electroendosmosis, EEO ≤ 0.12) films were formed on glass slides. For that purpose, a 1% (w/w) solution of agarose in deionized water was prepared, of which 400 µl were spread on the glass surface equilibrated at 40 °C on a heating plate. The agarose coated slide was then left to dry at 40 °C for 2 h, until formation of a dry agarose film.

To generate a lipid film on and inside the films of agarose, 30 µl of the lipid solution of interest were spread on the agarose using the procedure described in detail by Horger et al. (Horger et al., 2009). The sample was dried for 20 min under vacuum to remove residual chloroform, and subsequently immersed in a 0.1 mol l^−1^ glucose aqueous solution to allow simultaneous swelling of the agarose gel and of the vesicles. The dish remained undisturbed at room temperature for 3 h to yield the giant vesicles suspension. The vesicles were then immobilized in a 0.5% (w/v) agarose gel for imaging, as reported previously (Lira et al., 2016).

### 2.6 Hypo-osmotic treatment

We subjected cysts to osmotic shock by adding poly(ethylene glycol) (PEG) 2000 g mol^−1^ as a calibrated osmolyte (Money, 1989). The osmolyte was added to the zoospore suspension, directly after encystment by shaking, to a final concentration of 90 mmol l^−1^, to achieve an osmotic pressure of 0.6 MPa in the PEG solution as compared to water without PEG (Money, 1989). During encystment the CW is formed, and adding the osmolyte immediately after shaking means that the osmotic imbalance was applied on the cells as they were still devoid of a CW. The cyst-osmolyte mixture was applied to the PDMS substrate as a 80 µl droplet and left to incubate for 3 h. This time was chosen for the cells to equilibrate the pressure difference by producing internal osmolytes such as proline (Ambikapathy et al., 2002, Lew et al., 2004), and to build a complete CW. In this time frame the cells could still grow a germ tube, at a slower growth rate but without visible effect on the number of germination events, indicating that the initial osmotic treatment did not affect cell viability significantly. After this incubation period on the surface, 50 µl of the droplet were then exchanged four times with water, lowering the external osmotic pressure and creating a hypo-osmotic pressure imbalance (ΔP ∼ -0.6 MPa) between inside and outside the cysts. Staining with CWP-BDP or NR12S was performed right before applying the osmotic shock, as described above but using a 10 µmol l^−1^ dye solution in 90 mmol l^−1^ PEG 2000  g mol^−1^. Analysis of the effect of the osmotic shock was performed by looking at the cyst exclusively, as the germ tubes tips were moving too much to allow for fluorescence signal collection. Imaging was carried out right after shock, to minimize the effects of adaptation by the organism through osmotic regulation (Lew et al., 2004) or changes in enzyme activity (Ene et al., 2015). No time evolution in the CWP-BDP signal after placing the sample under the microscope was observed, within the imaging time frame (about 30 min). In parallel, a hyper-osmotic treatment involving the same PEG 2000  g mol^−1^ concentration, but added to the cells after 1 to 2 h after germination, was also attempted. This operation lead to almost instantaneous plasmolysis of the *Phytophthora* cells, accompanied by dye expulsion from the CW and internalization, making the imaging impossible.

### 2.7 Chemical treatments

To investigate the effect of chemical stress on the CW and PM properties, cells were treated with two different compounds reported to act on the structural properties of either the PM or the CW. Each treatment was started 1 h after application of the encysted zoospores on the substrate, and performed during 1 h before staining and imaging. During imaging, cells were kept in the same treatment conditions to avoid additional stress.

To perturb the CW composition and structure, valifenalate (Sigma Aldrich) solubilized in a 0.25 mmol l^−1^ stock solution in DMSO was added to the germlings to a final concentration of 125 nmol l^−1^ valifenalate (DMSO content < 0.05% (v/v)). To induce variations in PM composition and lipid order, fluopicolide (Sigma Aldrich) solubilized in a 0.26 mmol l^−1^ stock solution in DMSO was added to the germlings to a final concentration of 25 nmol l^−1^ fluopicolide (DMSO content < 0.05% (v/v)). To perturb the organized actin cytoskeleton of the pathogens, LatB solubilized in a 0.1 mmol l^−1^ stock solution in DMSO was added to the germlings to a final concentration of 1 µmol l^−1^ LatB and 1% (v/v) DMSO. To disrupt the microtubule network, oryzalin solubilized in a 0.1 mmol l^−1^ stock solution in DMSO was added to the germlings to a final concentration of 0.1 µmol l^−1^ oryzalin and 1% (v/v) DMSO. As a control, cells were also treated with 1% (v/v) DMSO only. Analysis of the effects of the chemical treatments was performed by looking at the hyphal tip exclusively.

### 2.8 Growth and invasivity assays

To assess whether staining with NR12S and CWP-BDP affects cells viability, we measured and compared the germ tube growth rate of cells in water, in a 10 µmol l^−1^ NR12S solution, or in a 10 µmol l^−1^ CWP-BDP solution. To do so, cells were stained right after encystment (CW not formed yet) and deposited on the liquid-free PDMS substrate without washing off the dye. A growth rate was computed by measuring the final germ tube length after 150 min growth.

We verified the invasion efficiency in the presence of the used chemicals, following the procedure adopted previously (Bronkhorst et al., 2021). The invasion efficiency is defined as the percentage of germinated cysts with germ tubes exceeding 10 µm in length, that have successfully fractured the artificial liquid-free PDMS surface 2 h post application (hpa). We used invasion efficiency as a functional evaluation of cell viability.

### 2.9 Statistical analysis

All the described imaging experiments, were repeated at least two times on different cell batches and different days (Table S1). For each repeat, at least ten cells were imaged. Fluorescence lifetime and intensity ratio distributions were extracted from the corresponding images, and summed over the different repeats. All distributions are built from at least 4000 data points. To ascertain the noise threshold for changes in lifetime or intensity ratio that can be detected with statistical significance, we performed measurements in homogeneous control media strictly deprived of spatial inhomogeneities (Figs. S22 & S23). We find a noise threshold, determined as the full-width at half-maximum (FWHM) of the observed distributions in homogeneous media, to be 0.45 ns for the lifetime measurements of CWP-BDP, and ratio = 0.02 for the ratiometric imaging of N12RS. We only consider an observed response significant if its change is either 1x or 3x larger than the noise floor for CWP-BDP and NR12S, respectively. The distribution widths we report in this paper on biological specimens are always well above those reported in homogeneous media; these thus do not represent measurement errors but rather reflect real spatial inhomogeneities in the CW or PM properties of the organism.

## 3 Results

### 3.1 Mechano-chemical response of walls to mechanical stress

In order to investigate structural modifications in the CW during non-invasive and invasive hyphal growth of *P.* infestans, we explored the feasibility of the wall-targeting fluorescent molecular rotor CWP-BDP that was successfully used to monitor CW modifications in Arabidopsis roots (Michels et al., 2020). Although the CWs of *Phytophthora* spp. have a different composition than plant CWs and contain mainly polymers of D-glucose (Mach, 2008; Meijer et al., 2006), we observe that also in *P. infestans* CWP-BDP binds to the CW, presumably through electrostatic interactions with other anionic carbohydrates (Fig. 1d & e). When implementing CWP-BDP in encysted zoospores, right after encystment, the germ tube growth rate is not affected (Fig. S24), suggesting that its presence does not impair cell viability nor CW integrity in the cells that germinate.

Using FLIM, we built three-dimensional reconstructions of the CW of *P. infestans* cells during non-invasive and invasive growth on an artificial host-mimicking substrate based on a PDMS elastomer (Bronkhorst et al., 2021)(Fig. 1d,e, Figs. S25-S27). The FLIM mesh size images provide quantitative information, which we extract as lifetime probability distributions (Fig. 1f & g). For example, in non-invasive cells, variations in lifetime emerge when comparing the cyst to the germ tube neck and tip. The lifetime diminishes locally to 2.0 ± 1.9 ns in the neck, while increasing slightly in the tip of the germ tube to 3.2 ± 0.9 ns (Fig. 1d & f). These differences reflect a relative increase in wall mesh size in the neck, and concomitant reduction in mesh size in the germ tube tip. By contrast, upon invasion, the fluorescence lifetime recorded within the tip of the germ tube in contact with the substrate rises strongly and abruptly to 3.7 ± 1.2 ns (Fig. 1e & g). The lifetime distribution keeps a comparable width but exhibits a small shoulder centered on the non-invasive case value of 3.2 ns. We postulate that this increase in lifetime, signalling a porosity reduction, is due to mechanical compression of the CW at the pathogen-substrate contact.

**Fig. 3:**
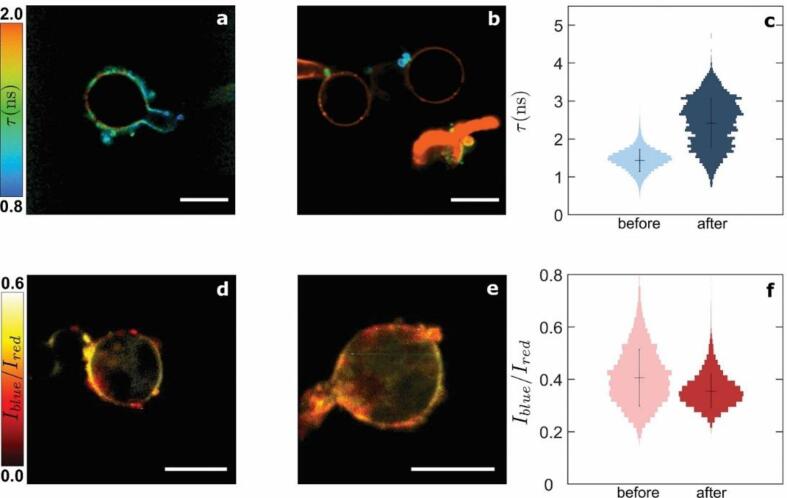
Effects of hypo-osmotic treatment on the cell wall and plasma membrane mechano-chemical properties of *Phytophthora infestans* germlings. Fluorescence lifetime cell wall mesh size map of germlings growing in (a) a 90 mmol l^−1^ PEG 2000  g mol^−1^ aqueous solution for 3 h, and (b) a 90 mmol l^−1^ PEG 2000  g mol^−1^ aqueous solution for 3 h followed by transfer to water. (c) Corresponding fluorescence lifetime probability distributions in (a-b) (N = 20 cells). Intensity ratio plasma membrane chemical polarity map of germlings growing in (d) a 90 mmol l^−1^ PEG 2000  g mol^−1^ aqueous solution for 3 h, and (e) a 90 mmol l^−1^ PEG 2000  g mol^−1^ aqueous solution for 3 h followed by transfer to water. (f) Corresponding intensity ratio probability distributions in (d-e) (N = 20 cells). Scale bars = 10 µm.

To confirm that the CW probe is indeed responsive to mechanical changes in CW porosity, we subjected the cells, stained with the same probe, to a hypo-osmotic stress. A hypo-osmotic stress leads to the tendency of the cell to inflate by water uptake. The resulting cell swelling is counteracted by the emergence of tensile stresses in the CW to re-establish tensegrity. As the amount of biomass in the CW is constant at these short time scales, these tensile stresses induce a compression of the polysaccharide network in the transverse direction. Consistently, a strong increase in lifetime is observed in the wall of cysts (Fig. 3a-c, Fig. S28). While the osmotic stress experiment induces a homogeneous tensile stress, and invasive growth a localized compressive stress, this experiment confirms that both contact and osmotic stresses, which are known to lead to mechanical stress in the CW, give rise to changes in CW porosity that can be revealed using the CWP-BDP probe. We note that it cannot be excluded that these sources of mechanical stress also result in the activation of biochemical pathways, e.g. those leading to the upregulation of CW remodelling enzymes as a protective mechanism to enhance CW rigidity and stress resistance (Ene et al., 2015). However, the contribution of these remodeling enzymes or other protectants is expected to be limited as we perform the imaging almost directly after stress application, whereas the activation and transcription of the encoding genes and the subsequent protein synthesis occurs over tens of minutes (Ene et al., 2015). These results illustrate that the CWP-BDP probe is capable of directly visualizing the locus of a mechanical stress.

### 3.2 Mechano-chemical response of membranes to mechanical stress

As a first attempt to investigate structural modifications in the PM of *P. infestans* we used a fluorescent molecular rotor named N+-BDP (Michels et al., 2020), with a conjugated structure and reporting mechanism identical to the one of CWP-BDP, but functionalized to target the PM. We previously used this PM probe successfully to monitor PM modifications in Arabidopsis roots (Michels et al., 2020). Staining of the PM in *P. infestans* was also successful but its dynamic response range to changes in membrane tension was not suitable to clearly highlight variations in membrane structural properties. We then switched to the chemical polarity probe NR12S developed by Kucherak et al. (Kucherak et al., 2010) (Fig. 2a). We implemented NR12S in encysted zoospores, right after encystment, and showed that germ tube growth rate is not affected by the staining (Fig. S24), suggesting that the presence of NR12S does not impair cell viability nor PM integrity in the cells that germinate.

To unravel how growth and invasion affect the properties of the PM that lies underneath the CW, we compare the response of the probe NR12S between non-invasive versus invasive hyphal growth. We record the emission intensity in cells stained with the NR12S probe within two channels, and represent the results as intensity ratio images from which we derive probability distributions (Fig. 2d & e). In cells growing non-invasively, the intensity ratio remains constant throughout the cell, around 0.4. This suggests that the PM composition and hydration level are relatively constant along the germ tube at early stages of growth. The invasive part of cells could not be imaged with NR12S due to the low dye penetration and/or emission intensity underneath the PDMS surface. We explored various possibilities to resolve this. We attempted to introduce the stain both before and after tip penetration into the substrate, which yielded the same result. We also attempted invasion experiments on clear agar medium. However, cells showed a lack of adhesion to the agar surfaces, which lead to a complete suppression of invasive growth. In parallel, hypo-osmotic stress only results in small changes in the ratiometric measurements of the PM (Fig. 3d & f). This implies that the PM polarity and lipid order is only weakly sensitive to the compressive forces that push the PM against the CW upon cell swelling. This observation suggests that upon invasive growth, as the hyphal tip is mechanically stressed by the substrate, compressive forces would not substantially alter the properties of the membrane. What causes the lack of fluorescence signal coming from the hyphal tip during invasive growth remains unclear at this point.

In mature *P. infestans* mycelium, the intensity ratio is decreased to 0.22 ± 0.15 (Fig. 2f & g), in comparison to the ratio of 0.42 ± 0.21 measured in the tip of young germ tubes. This observation suggests a significant change in PM composition and lipid order as the cells grow and maturate, and confirms the probe responsiveness to changes in membrane chemical polarity. We validated this hypothesis by implementing NR12S in synthetic giant-unilamelar vesicles made of DOPC, SM, and cholesterol (1:1:0.7 M ratio). This lipid composition can lead to phase separation between an ordered (Lo) phase rich in SM and cholesterol, and a liquid-like (Ld) phase, enriched in DOPC (Fig. 2h). GUVs made of only DOPC and SM show a single phase, and a narrow intensity ratio distribution centered around 0.17 ± 0.11. In the cholesterol-supplemented GUVs, phase separation and the global increase in lipid order is reflected by an upward shift of the ratio distribution mean value and width to 0.26 ± 0.22 (Fig. 2i). These results confirm the responsiveness of NR12S to changes in lipid phase. The difference in PM properties in the mycelium as compared to the cyst life stage thus hints at a reduced lipid order and packing density in mycelial membranes. We speculate that these differences are due to changes in PM composition, e.g. lipid composition and/or protein content, as both life stages utilize distinct metabolic pathways in which lipids, and hence the PM, play different roles (Grant et al., 1988, Savidor et al., 2008, Pang et al., 2017, Rodenburg et al., 2018).

### 3.3 Structural modifications in the cell wall and plasma membrane by chemical control agents

**Fig. 4:**
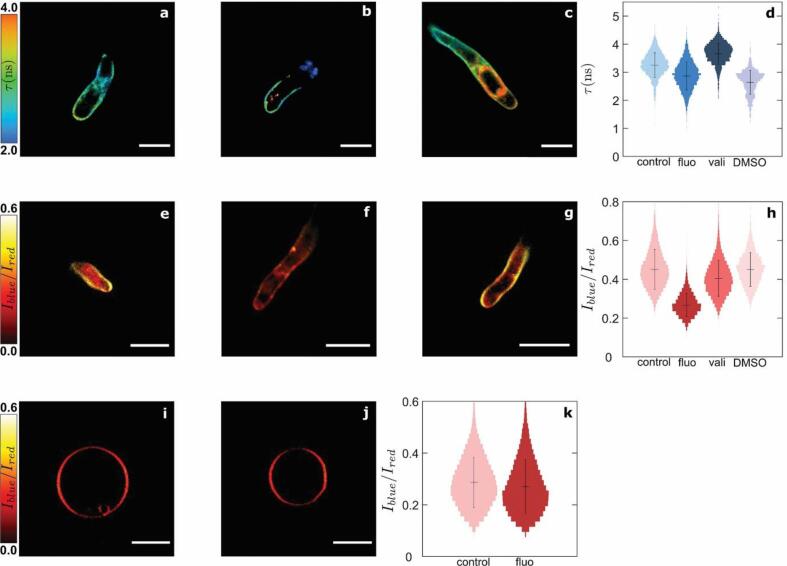
Effects of treatments with fluopicolide and valifenalate on the cell wall and plasma membrane mechano-chemical properties of *Phytophthora infestans* germlings. Fluorescence lifetime cell wall mesh size map of germlings growing in (a) water for 1 h, (b) water for 1 h followed by treatment with 25 nmol l^−1^ fluopicolide (fluo) for 1 h, and (c) water for 1 h followed by treatment with 125 nmol l^−1^ valifenalate (vali) for 1 h. (d) Fluorescence lifetime probability distributions in (a-c) (N = 30 cells). Intensity ratio plasma membrane chemical polarity map of germlings growing in (e) water for 1 h, (f) water for 1 h followed by treatment with 25 nmol l^−1^ fluopicolide (fluo) for 1 h, and (g) water for 1 h followed by treatment with 125 nmol l^−1^ valifenalate (vali) for 1 h. (h) Intensity ratio probability distributions in (e-g) (N = 30 cells). Intensity ratio chemical polarity map of DOPC:SM:Cholesterol = 1:1:0.7 (molar ratio) synthetic vesicles in (i) absence and (j) presence of 25 nmol l^−1^ fluopicolide (fluo). (k) Fluorescence lifetime probability distributions in (i-j) (N = 30 cells). Scale bars = 10 µm.

Using the CW and PM molecular nanosensors, we investigated the effects of different chemical stresses on *P. infestans* during cyst germination on PDMS elastomer surfaces. In particular, we looked at the effect of adding two active ingredients from oomicides, i.e. valifenalate and fluopicolide (Fig. 4), to evaluate if CWP-BDP and NR12S could resolve their site of action. At high doses these compounds decrease *Phytophthora* cell viability culminating in cell death (Saville et al., 2015, Wu et al., 2019). Since this is associated with a variety of stress responses, we performed the treatments at sub-lethal doses, as verified by our invasivity measurements. This allows us to observe changes in CW or PM integrity without taking into account the effect of additional stress responses.

We selected the PM targeting compound fluopicolide and the cellulose synthase inhibitor valifenalate (FRAC, 2021) to verify their specifity of action to either membranes or walls. Fluopicolide dislocates spectrin-like proteins in *Phytophthora* from the membrane to the cytoplasm (Toquin et al., 2007, Toquin et al., 2009). Upon treatment with 25 nmol l^−1^ fluopicolide, NR12S indicates a significant drop in the NR12S intensity ratio within the PM, going from 0.42 ± 0.21 to 0.23 ± 0.09 (Fig. 4e,f,h, Fig. S29). This change could reflect either a loss in PM tensegrity by spectrin-like protein expulsion and potential cytoskeletal de-adhesion from the membrane, or result from the intercalation of the rather polar fluopicolide molecule into the PM. To discriminate between these two hypotheses, we performed experiments in synthetic vesicles which lack spectrins and other PM proteins, by submitting GUVs to the same concentration of fluopicolide (Fig. 4i–k, Fig. S30). No change in intensity ratio was reported in synthetic vesicles, which confirms that the increase in polarity within the PM is due to an alteration of the membrane structure and not by fluopicolide entry into the PM. By contrast, the same fluopicolide treatment results in only minor changes in the CW, as confirmed by CWP-BDP fluorescence lifetime imaging (Fig. 4a,b,d, Fig. S29), likely influenced by the presence of DMSO (Fig. 4d, Fig. S31). This suggests that there is no direct link between the correct localization of spectrin-like proteins in the PM and wall mechanics and biosynthesis. We note that while NR12S displays a significant shift in intensity ratio upon treatment with this low fluopicolide concentration, this dose does not lead to a significant inhibition of pathogen invasion. In the absence of fluopicolide the invasivity is 71.3% (N = 138 cells), versus 63% (N = 84 cells) with fluopicolide. This indicates that NR12S is sensitive enough to report changes in the physico-chemical properties of the cells well before cell functional viability is hampered.

Valifenalate has been used to target and weaken the CW of oomycetes (Marsilii et al., 2009, Salamone et al., 2013). We observe that valifenalate distinctly changes the CW properties in *P. infestans*; imaging with CWP-BDP reveals an increase in fluorescence lifetime from 3.2 ± 0.9 ns in non-treated germlings to 3.6 ± 0.8 ns in germlings treated with 125 nmol l^−1^ valifenalate, and the emergence of distinct spatial variations along the germ tube, marking the transition between the wall portions synthesized before and during treatment (Fig. 4a,c,d, Fig. S32). Staining of an unknown structure inside the cells led to an intracellular fluorescent signal that was excluded from the analysis. This structure could result from the accumulation of CW precursors in the cytoplasm. Valifenalate treatment results in a denser CW, which not only changes the probe lifetime but also weakly reduces the staining efficiency due to a lower permeability. We previously reported a similar trend in the evolution of the wall mesh size during polarized growth of *Arabidopsis* root hairs (Michels et al., 2020). During root hair growth the CW initially consists of a dense network of flexible carbohydrates, malleable enough to mechanically yield under turgor to enable tip growth, but later on the CW is reinforced by a network of stiff cellulose fibers which exhibit a much larger mesh size. Similarly, in the case of *P.* infestans, the absence of the cellulose network results in a decrease in porosity and an increase in fluorescence lifetime of the probe. Our observations are consistent with a cellulose synthesis inhibition induced by valifenalate treatment, which results in less porous and more malleable CWs. By comparison, the intensity ratio of NR12S is not affected, showing no influence of valifenalate on PM properties (Fig. 4e,g,h, Fig. S32). Also for this compound, the effects on CW and PM are decoupled and the chemical acts specifically on a singular target in the CW-PM continuum. When investigating cell viability under treatment with 125 nmol l^−1^ valifenalate, we only notice a slight reduction in the invasivity from 71.3% (N = 138 cells) to 59.2% (N = 103 cells). This confirms the sensitivity of CWP-BDP to changes in CW properties even before large effects on cell functional viability can be detected.

### 3.4 Changes in wall and membrane in response to cytoskeletal disruption

The cell cytoskeleton is known to play a leading role in establishing and maintaining the polarity required for hyphal growth and thereby in the invasion fitness of *Phytophthora* pathogens (Heath and Kaminskyj, 1989, Temperli et al., 1991, Hyde and Hardham, 1993, Geitmann and Emons, 2000, Blum et al., 2010, Ketelaar et al., 2012, Bronkhorst et al., 2021). To examine how the CW-PM continuum of *P. infestans* responds to perturbations in cytoskeletal organization, we studied the consequences of treatment with the actin depolymerizing drug LatB (Ketelaar et al., 2012) and the microtubule polymerization inhibitor oryzalin (Morejohn et al., 1987, Hyde and Hardham, 1993, Dostál and Libusová, 2014).

**Fig. 5:**
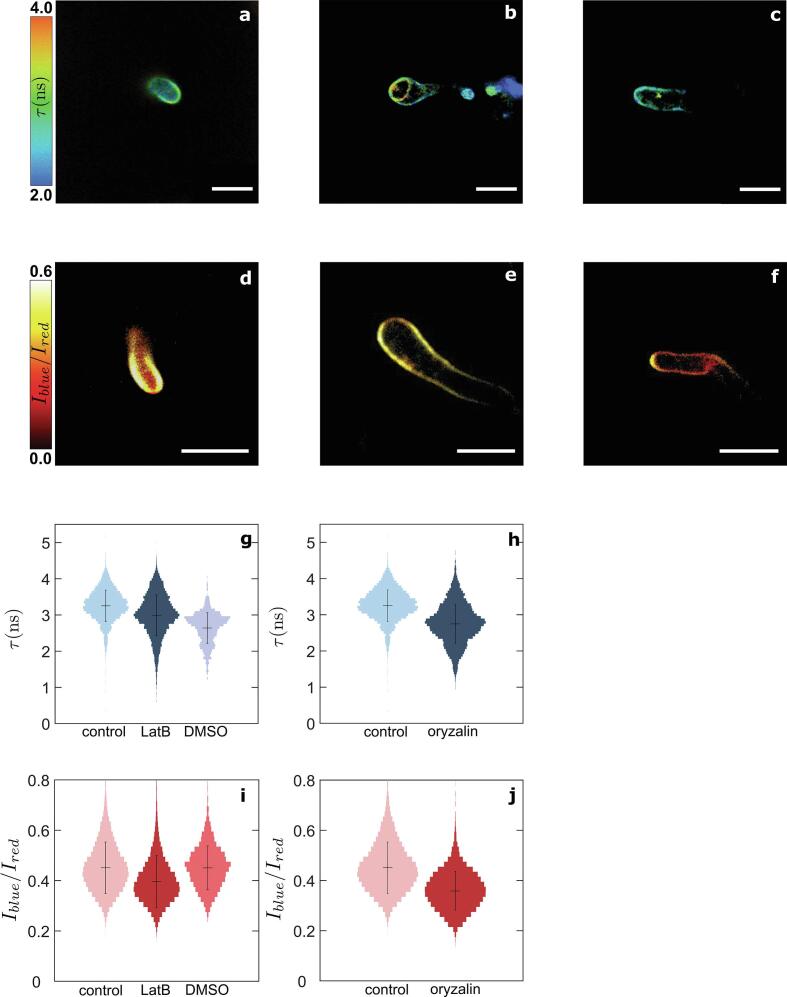
Effects of treatments with cytoskeletal depolymerizing drugs on the cell wall and plasma membrane mechano-chemical properties of *Phytophthora infestans* germlings. Fluorescence lifetime cell wall mesh size map of germlings growing in (a) water for 1 h, (b) water for 1 h followed by treatment with 1 µmol l^−1^ latrunculin B for 1 h, and (c) water for 1 h followed by treatment with 0.1 µmol l^−1^ oryzalin for 1 h. Intensity ratio plasma membrane chemical polarity map of germlings growing in (d) water for 1 h, (e) water for 1 h followed by treatment with 1 µmol l^−1^ latrunculin B for 1 h, and (f) water for 1 h followed by treatment with 0.1 µmol l^−1^ oryzalin for 1 h. (g, h) Fluorescence lifetime probability distributions in (a-c) (N = 30 cells). (i, j) Intensity ratio chemical polarity probability distributions in (d-f) (N = 30 cells). Scale bars = 10 µm.

Treatment with LatB at 1 µmol l^−1^ affects the morphology of the cells, which exhibit the tip swelling behavior characteristic for the loss of polarization. However, very minor fluorescence lifetime changes of CWB-BDP are visible within the CW (Fig. 5a,b,g, Fig. S33). A shift in intensity ratio of NR12S from 0.42 ± 0.21 to 0.36 ± 0.17 is visible within the PM (Fig. 5d,e,i, Fig. S33). Higher doses of LatB strongly disrupt the actin cytoskeleton involved in the transport of CW precursors to the growing tip (Ketelaar et al., 2012; Meijer et al., 2014, Kots et al., 2017). Even though one could have expected that 1 µmol l^−1^ of LatB would affect CW synthesis, our images reveal an intact CW for all cells studied under this treatment. Hence we conclude that LatB treatment does not substantially affect the CW. The same treatment results in an increase in the PM polarity, possibly reflecting a reduction of membrane stability due to the lack of tension applied by the disrupted cytoskeleton.

Treatment with the microtubule inhibitor oryzalin at 0.1 µmol l^−1^, leads to a reduction of fluorescence lifetime of CWP-BDP to 2.5 ± 0.9 ns in the hyphal tip (Fig. 5a,c,h, Fig.S34), while the intensity ratio of NR12S decreases to 0.34 ± 0.18 (Fig. 5d,f,j, Fig. S34). While the exact function of microtubules in germinated cysts, both during invasive and non-invasive growth, is yet to be resolved, our results show that disruption of the microtubule network leads to changes in both the CW porosity and PM tensegrity. This could point towards a role for microtubules in regulating the structure and tension in both organs. At this low dose of oryzalin, the invasivity of the cells remains at 68.1% (N = 201 cells). Also here, the combination of CWP-BDP and NR12S to map CW porosity and PM integrity enables connecting action on the cytoskeleton to their effects on the PM-CW continuum, even when cell viability is not affected.

## 4 Discussion and conclusions

In walled cells the CW and PM form a continuum that plays a major role in mechano-chemical responses. The CW being the stiffest part of the cell, and the direct connection between the cell and its immediate surroundings, is the site where the perception and transduction of mechanical cues begin. In this study we exploited molecular sensors to show that mechanical interactions with a substrate elicit substantial reductions in the cell wall porosity, presumably due to the mechanical squeezing of water from the wall at the pathogen-substrate contact zone. This scenario of intimate mechanical contact between a pathogen and a stiff substrate is also encountered during host entry by pathogens into plant hosts. We recently uncovered that host entry by *Phytophthora* pathogens occurs through a specialized mechanism of invasive tip growth, called the naifu mechanism (Bronkhorst et al., 2021). How naifu invasion is regulated and how information about the state of stress at the point of contact is internalized by the cell is currently unknown. Given that naifu invasion is critical to successful host entry and thus for the life cycle of these pathogens (Bronkhorst et al., 2021), it stands to reason that mechanical feedback into the cell interior, e.g. to regulate cytoskeletal architecture and cell wall properties to accomodate the large stresses at the point of contact, could be an important aspect that remains to be explored. The tools presented here offer a way to visualize local mechanical stress effects in the CW and PM, and correlate these to the intracellular response. For example, the fact that we observe substantial changes in CW properties under mechanical stress could hint to a possible role for CW mechanosensors in this process. While the molecular mediators of mechano-perception in *Phytophthora* are still unknown, various CW-localized mechano-receptors have been identified in other walled microorganisms, such as yeast (Kock et al., 2015, Elhasi and Blomberg, 2019, Neeli-Venkata et al., 2020). The molecular sensors presented in this paper enable us to experimentally track mechanical-based processes in *Phytophthora* and related plant pathogens, and could be instrumental in correlating the downstream effects of wall mechanosensor activation to the local state of stress.

In agriculture, chemical control is a crucial component of integrated disease management practices. To date, the development of oomicides that inhibit growth of the pathogen and combat *Phytophthora* diseases has relied partly on compounds able to prevent structural adaptation, or that induce structural modifications as a side effect (Blum et al., 2010, Gisi and Sierotzki, 2015). In particular, CW integrity has been identified as a critical feature of pathogenicity (Brus-Szkalej et al., 2021), and CW disruption is a potential target of interest. The mechano sensors presented here offer a new way to gain insights into the mode-of-action of CW- and PM-targeting agrochemicals, and can thus support efforts that aim to inhibit host entry and/or pathogenicity. In this study, we implemented the CW-targeting molecular rotor CWP-BDP and the PM ratiometric probe NR12S in *P. infestans*, but these molecular sensors are readily applicable to other oomycetes. We also tried to implement fluorescent molecular rotors that successfully target the cytoplasm or the vacuole in plant cells (Michels et al., 2020). Unfortunately the dyes did not penetrate the hyphae at all, highlighting the lower permeability of oomycete cells in comparison to plant root cells.

By combining the two fluorescent mechano sensors, we are able to map variations in CW mesh size and PM chemical polarity during growth and invasion. Upon treatment with active ingredients of oomicides, or with cytoskeletal depolymerizing drugs, clear changes in CW and PM structural properties are visible and give a real-time indication on the effect of the chemical stress. To achieve a quantitative measurement, systematic calibrations of the two molecular sensors are needed. These are not trivial steps as they need to be conducted in a medium that is simultaneously representative for the chemical composition of the compartment of interest, regarding both polarity and length scales, and quantitatively tuneable. For example, to have a reference in terms of CW mesh size, we could imagine implementing CWP-BDP in a synthetic polysaccharide network with controlled mesh sizes and polymer charge densities. In parallel, to determine the PM chemical polarity and lipid phase, we envision systematic studies of the NR12S response in vesicles reconstituted from cell extracts and systematically introducing sterols in the preparation protocol as a way to tune the lipid order. These calibrations can be challenging, as the CW and PM compositions are highly complex and vary from species to species, cell to cell and location to location. Yet, even without calibration to enable quantitative data, the molecular sensors already provide valuable qualitative insights into mechano-chemical heterogeneities, reflecting relative changes in probe confinement and local chemical polarity, respectively, as well as providing information on the dynamics of cellular processes at a high resolution that cannot currently be achieved by other means. The mechanosensors could be valuable in characterizing the phenotypes of mutants in which target genes are disrupted by CRISPR-CAS mediated gene editing and silenced by RNA interference, and as such help in deciphering the role of different biochemical pathways and proteins in determining CW and PM structural properties.

Unravelling the complex mechano-chemical interactions between pathogens and host would ultimately require implementation of this method on real plant hosts rather than on artificial surfaces, to take into account the complex mechano-biological responses of both parties. Chemical engineering of the mechanosensors to provide even more precise, and organism specific, targeting, and using designs with two complementary emission wavelengths, would allow simultaneous mapping of both pathogen and plant properties, and help understand their mechanical interactions. To do so, several promising strategies to achieve selective binding arise; for instance, the use of metabollic labelling, previously employed to incorporate alkyne or azide groups in the pectin network of the *Arabidopsis* CW, allowing for a subsequent click-reaction to fluorescent dyes (Anderson et al., 2012, Dumont et al., 2016, Zhu et al., 2016), or the use of genetic labelling by means of SNAP-tags, previously implemented in animal cells to label e.g. PM proteins (Sun et al., 2011, Bosch et al., 2014). Such approaches could prove valuable for a more systemic perspective on the complex mechanobiology and mechanochemistry of host invasion.

## CRediT authorship contribution statement

**Lucile Michels:** Conceptualization, Data curation, Formal analysis, Investigation, Methodology, Project administration, Resources, Software, Visualization, Writing. **Jochem Bronkhorst:** Conceptualization, Data curation, Formal analysis, Investigation, Methodology, Project administration, Resources, Software, Visualization, Writing. **Michiel Kasteel:** Investigation, Methodology, Resources, Validation. **Djanick de Jong:** Investigation, Methodology, Resources, Validation. **Bauke Albada:** Investigation, Methodology, Resources, Validation. **Tijs Ketelaar:** Conceptualization, Funding acquisition, Investigation, Resources, Supervision, Validation. **Francine Govers:** Conceptualization, Funding acquisition, Investigation, Resources, Supervision, Validation, Writing - review & editing. **Joris Sprakel:** Conceptualization, Data curation, Funding acquisition, Investigation, Methodology, Project administration, Resources, Supervision, Validation, Writing.

## Declaration of Competing Interest

The authors declare that they have no known competing financial interests or personal relationships that could have appeared to influence the work reported in this paper.
