# Supplementary material for: Molecular sensors reveal the mechano-chemical response of Phytophthora infestans walls and membranes to mechanical and chemical stress
Source: Cell Surf. 2022 Jan 7;8:100071. doi: 10.1016/j.tcsw.2021.100071 (PMC8760408; doi:10.1016/j.tcsw.2021.100071)
Supplement: Supplementary data 1 [file mmc1.docx]

## Supporting Information

Article title: Molecular sensors reveal the mechano-chemical response of *Phytophthora infestans* walls and membranes to mechanical and chemical stress

Authors: Lucile Michels*, Jochem Bronkhorst*, Michiel Kasteel, Djanick de Jong, Tijs Ketelaar, Francine Govers & Joris Sprakel

The following Supporting Information is available for this article:

**
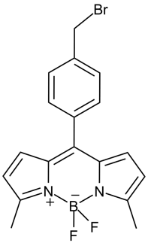

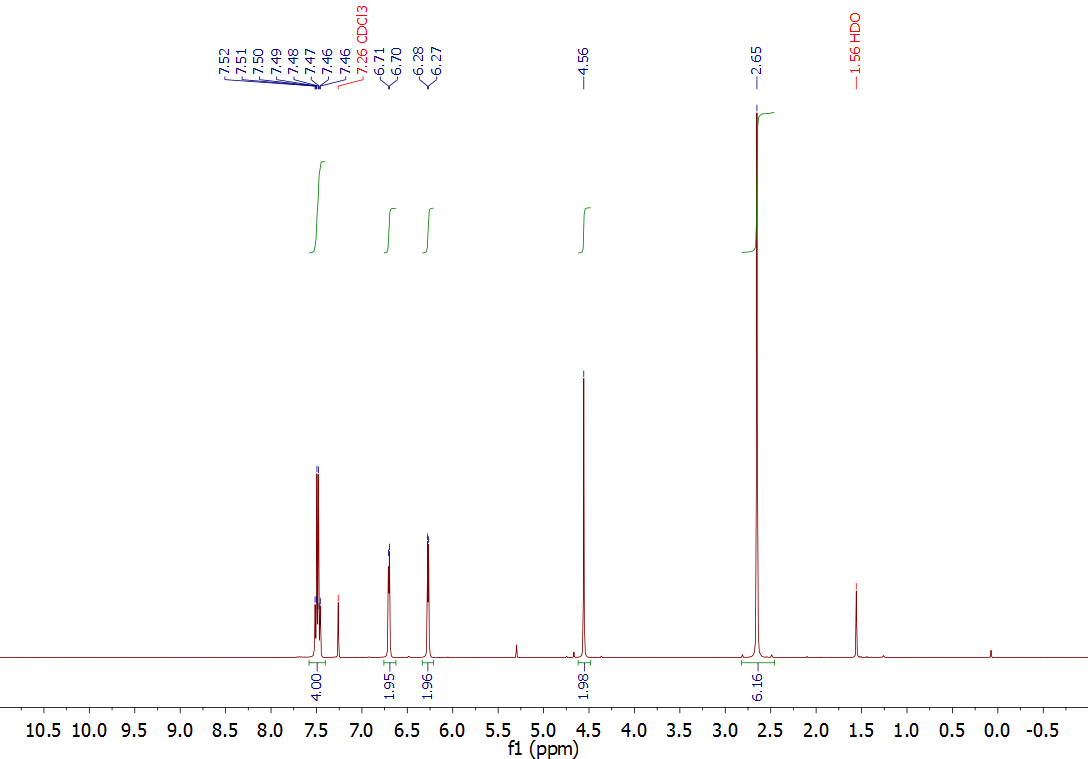
**

**Fig. S1** **^1^H NMR spectrum of 10-(4-(bromomethyl)phenyl)-5,5-difluoro-3,7-dimethyl-5H-dipyrrolo [1,2-c:2',1'-f] [1,3,2] diazaborinin-4-ium-5-uide (1)**

**
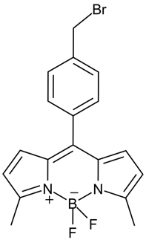

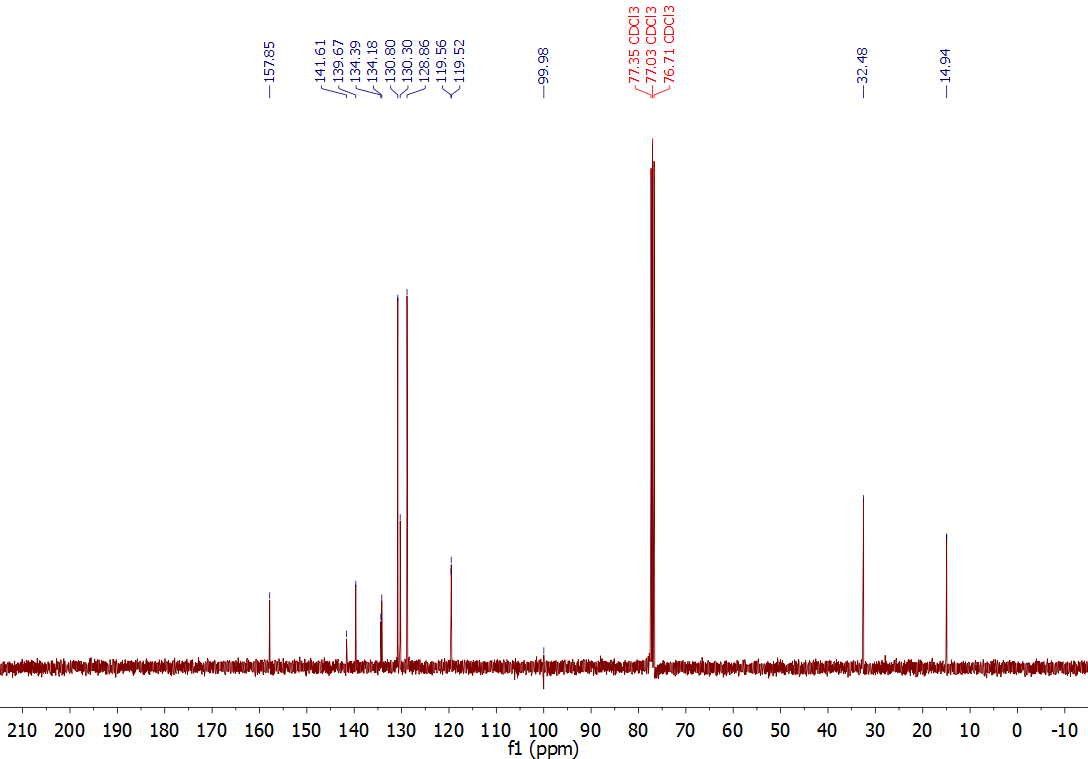
**

**Fig. S2 ^13^C NMR spectrum of 10-(4-(bromomethyl)phenyl)-5,5-difluoro-3,7-dimethyl-5H-dipyrrolo [1,2-c:2',1'-f] [1,3,2] diazaborinin-4-ium-5-uide (1)**

**
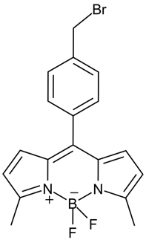
**
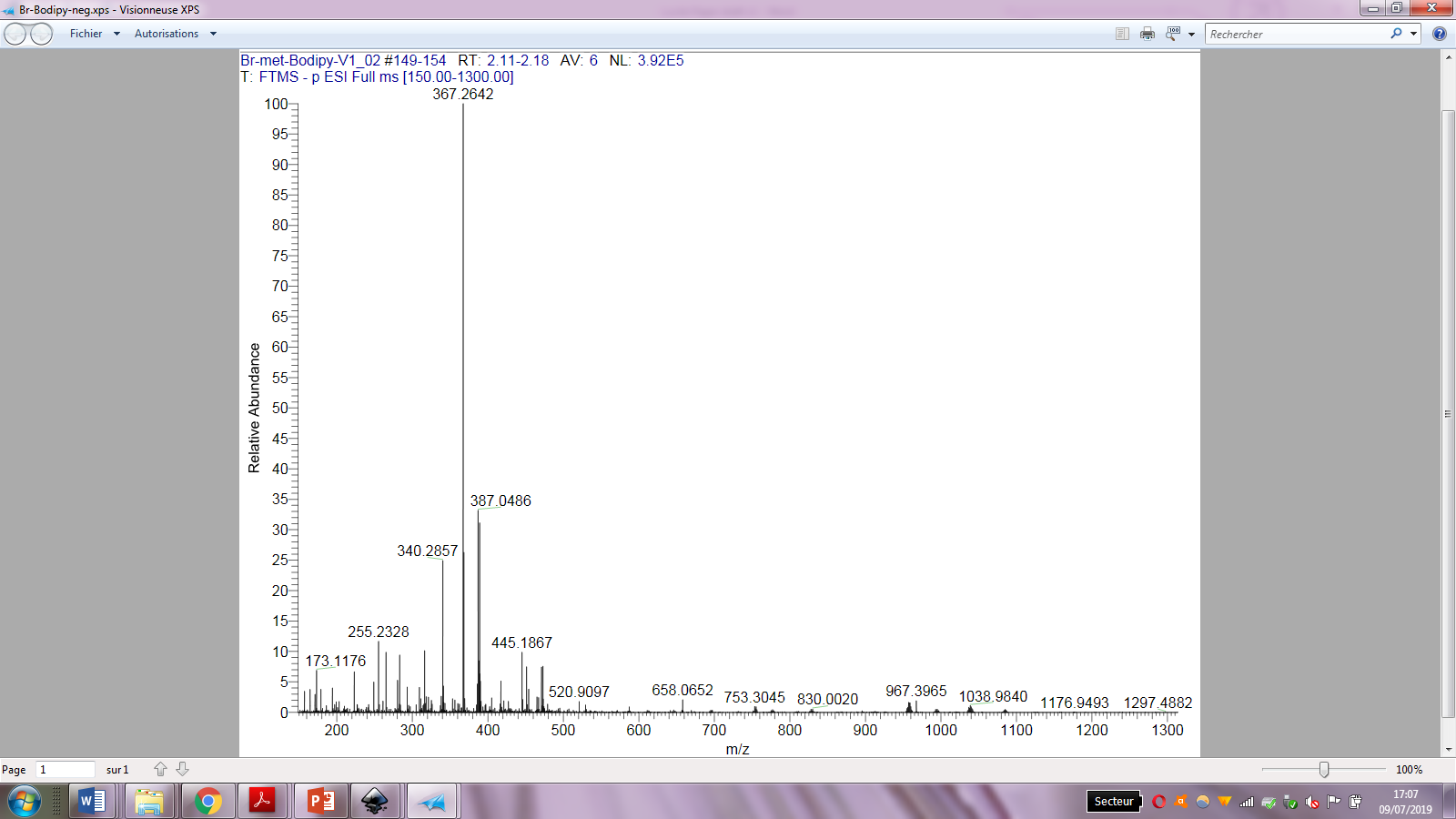


**Fig. S3 HRMS (ESI) spectrum of 10-(4-(bromomethyl)phenyl)-5,5-difluoro-3,7-dimethyl-5H-dipyrrolo [1,2-c:2',1'-f] [1,3,2] diazaborinin-4-ium-5-uide (1)**

**
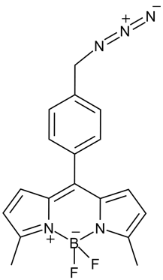

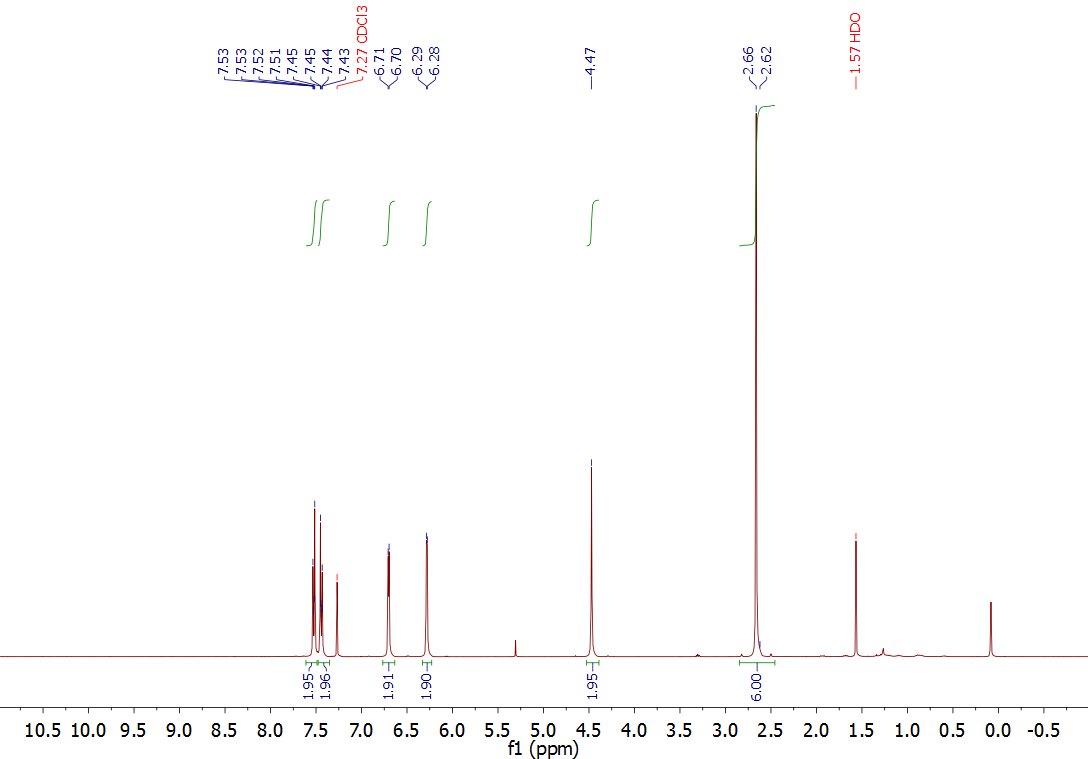
**

**Fig. S4 ^1^H NMR spectrum of 10-(4-(azidomethyl)phenyl)-5,5-difluoro-3,7-dimethyl-5H-dipyrrolo [1,2-c:2',1'-f] [1,3,2] diazaborinin-4-ium-5-uide (2)**

**
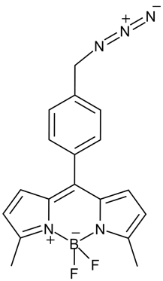

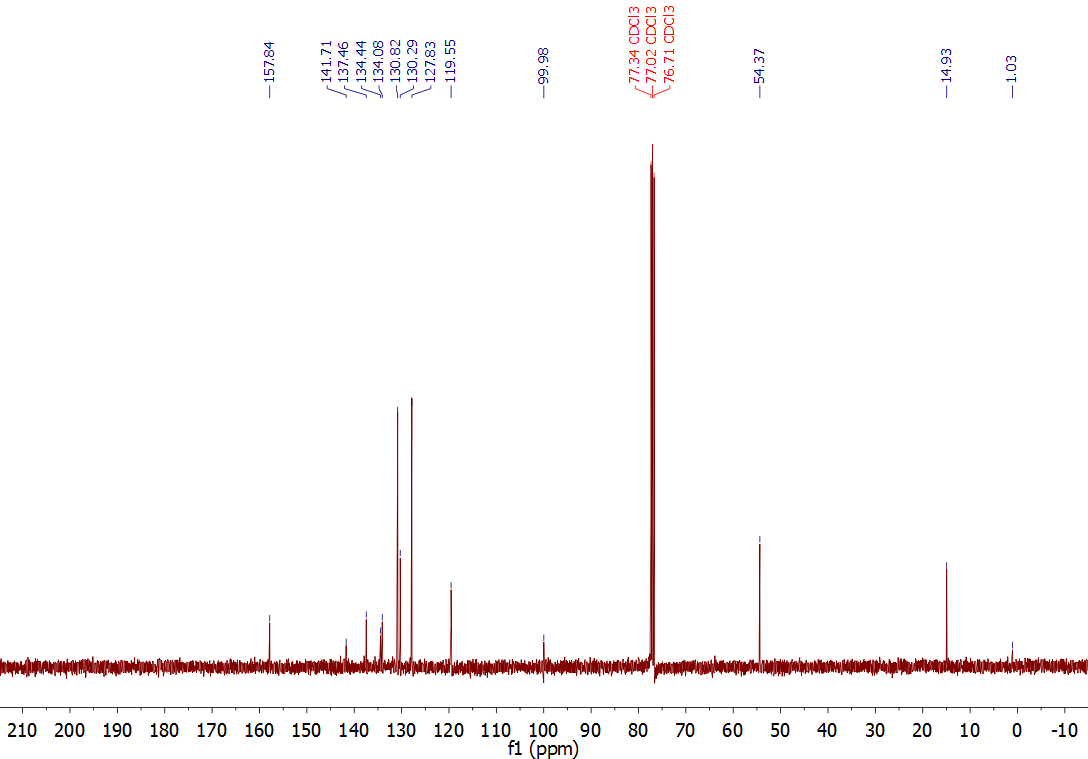
**

**Fig. S5 ^13^C NMR spectrum of 10-(4-(azidomethyl)phenyl)-5,5-difluoro-3,7-dimethyl-5H-dipyrrolo [1,2-c:2',1'-f] [1,3,2] diazaborinin-4-ium-5-uide (2)**

**
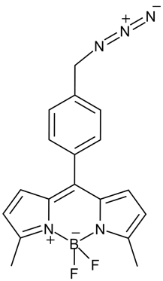
**
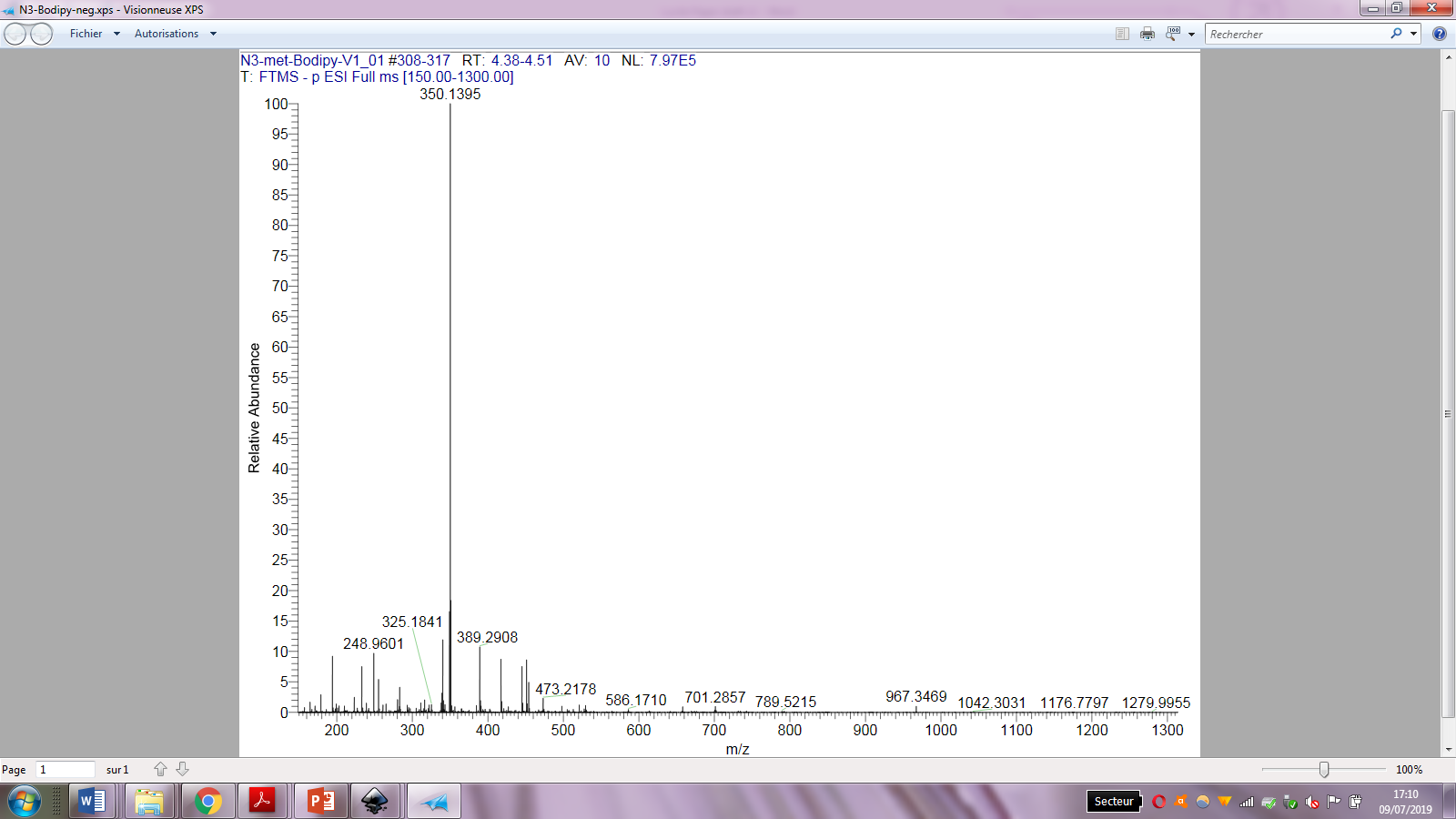


**Fig. S6 HRMS (ESI) spectrum of 10-(4-(azidomethyl)phenyl)-5,5-difluoro-3,7-dimethyl-5H-dipyrrolo [1,2-c:2',1'-f] [1,3,2] diazaborinin-4-ium-5-uide (2)**

**

**
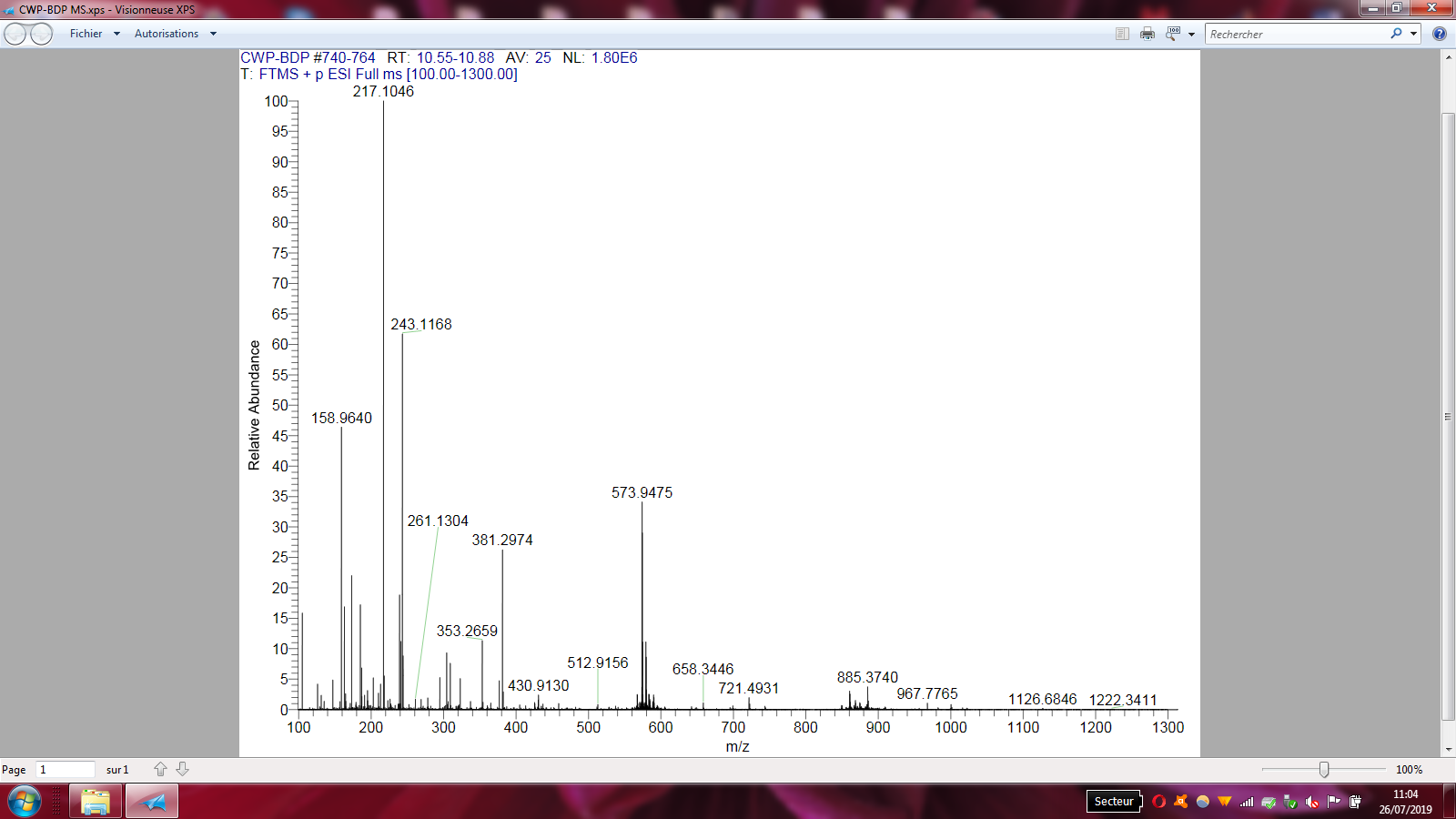


**Fig. S7 HRMS (ESI) spectrum of CWP-BDP**


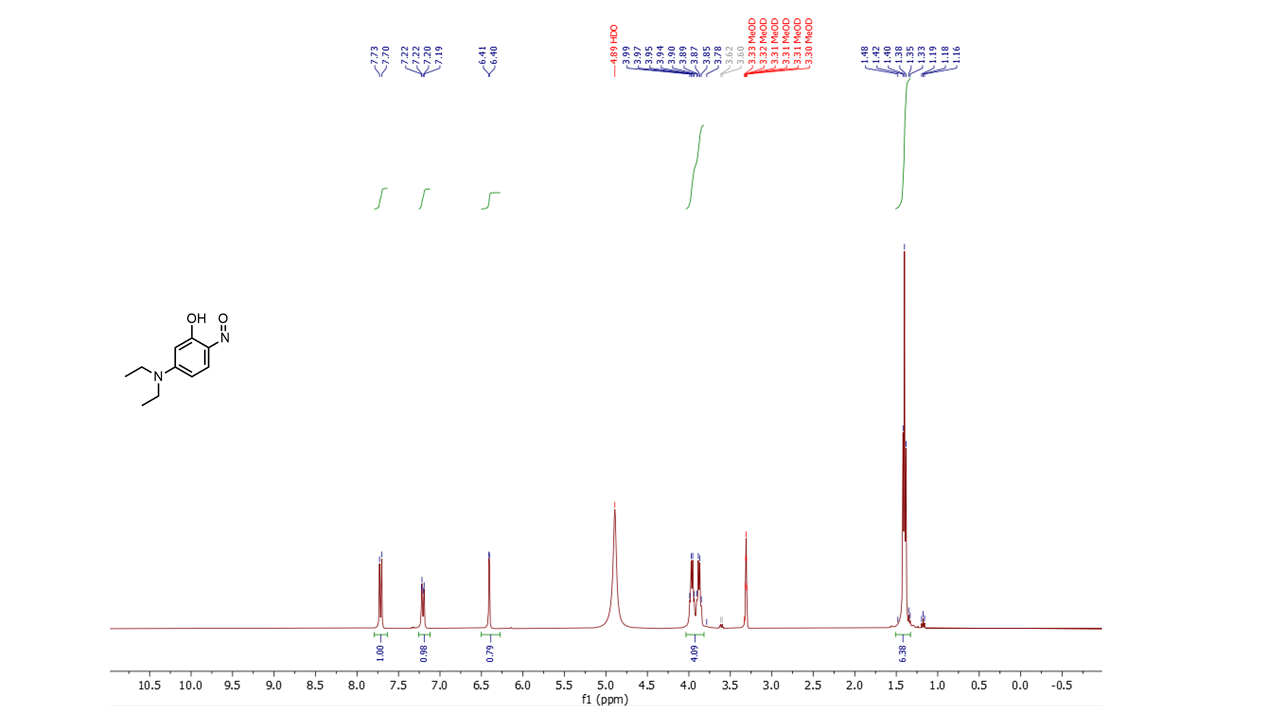


**Fig. S8 ^1^H NMR spectrum of 5-(Diethylamino)-2-nitrosophenol (4)**


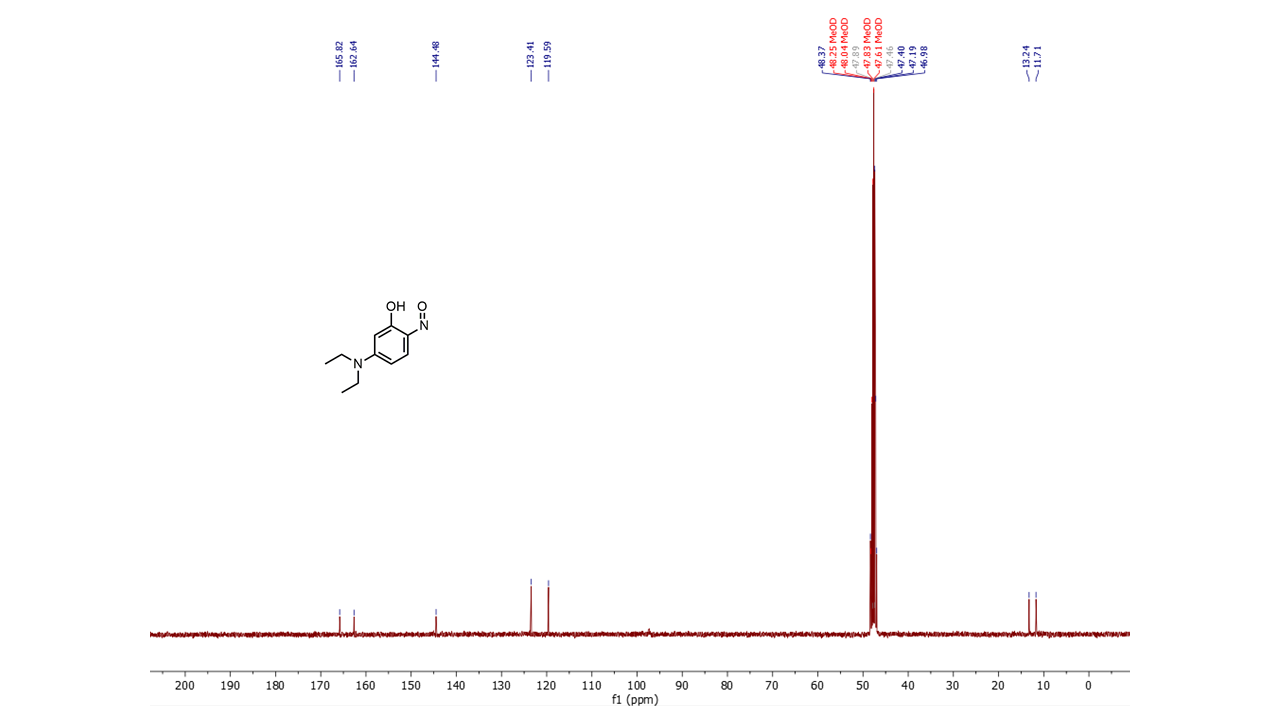


**Fig. S9 ^13^C NMR spectrum of 5-(Diethylamino)-2-nitrosophenol (4)**


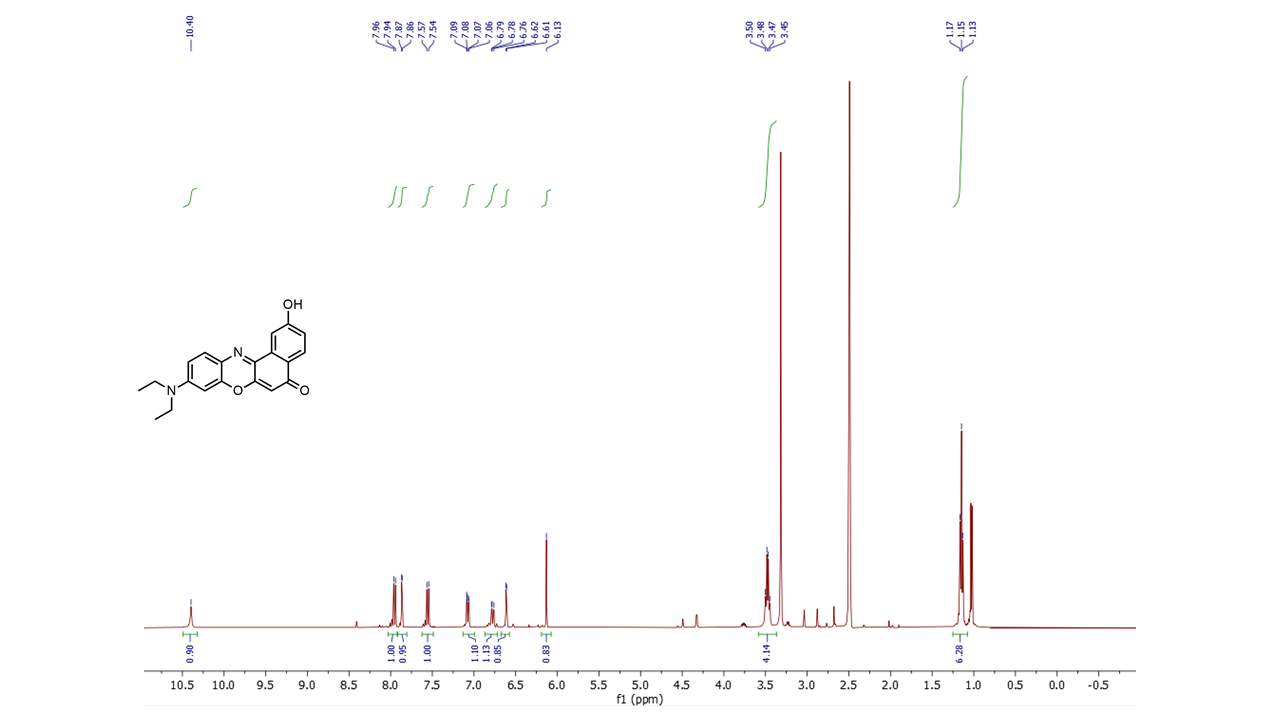


**Fig. S10 ^1^H NMR spectrum of 9-(Diethylamino)-2-hydroxy-5H-benzo[a]phenoxazin-5-one (5)**


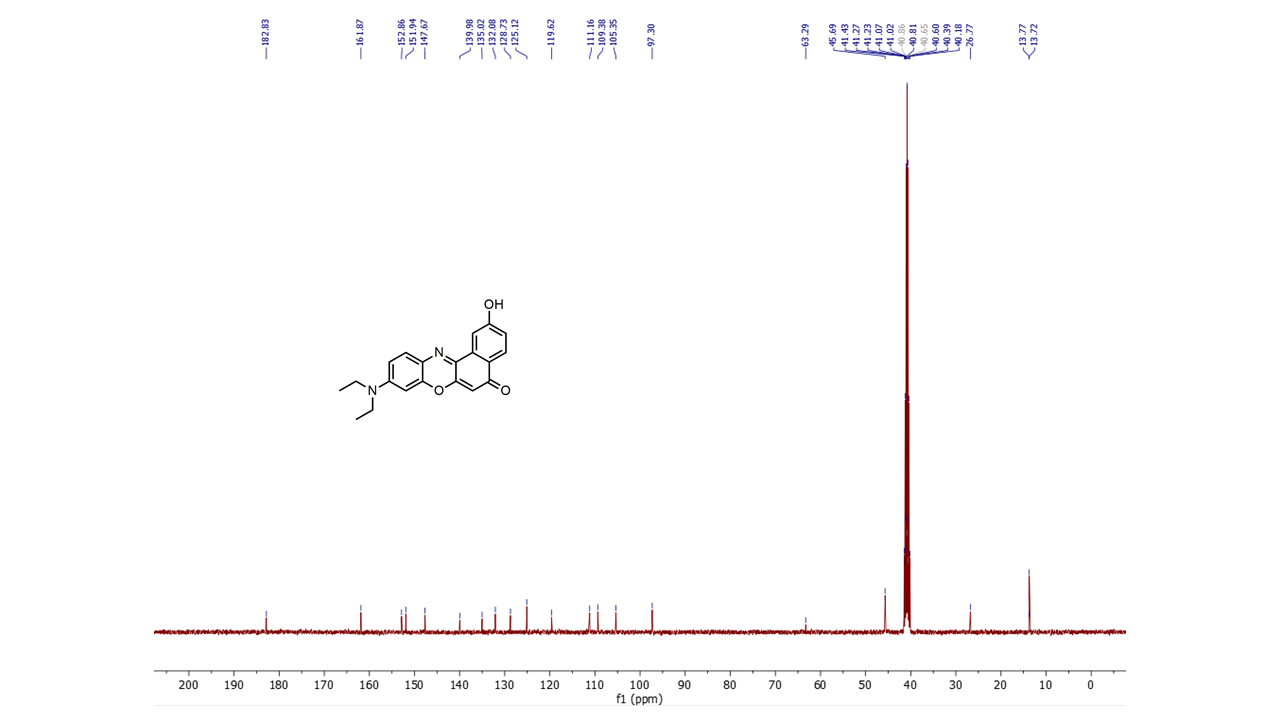


**Fig. S11 ^13^C NMR spectrum of 9-(Diethylamino)-2-hydroxy-5H-benzo[a]phenoxazin-5-one (5)**


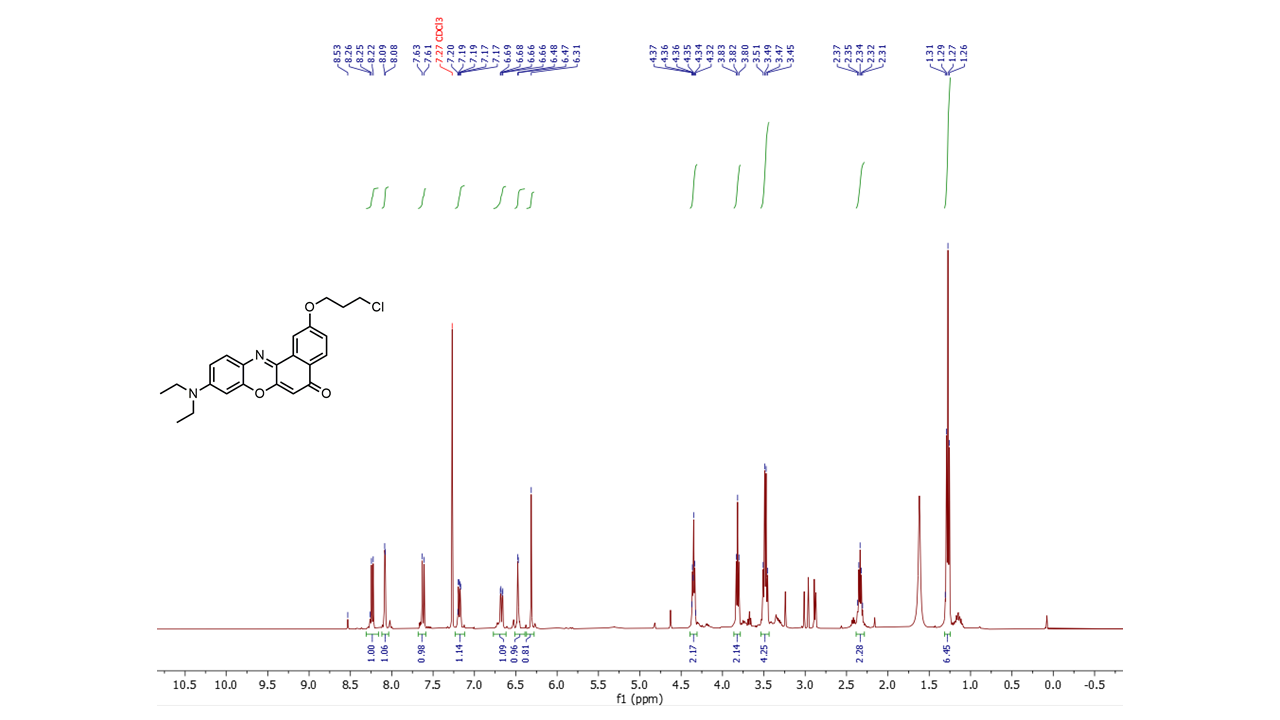


**Fig. S12 ^1^H NMR spectrum of 2-(3-Chloropropoxy)-9-(diethylamino)-5H-benzo[a]phenoxazin-5-one (6)**


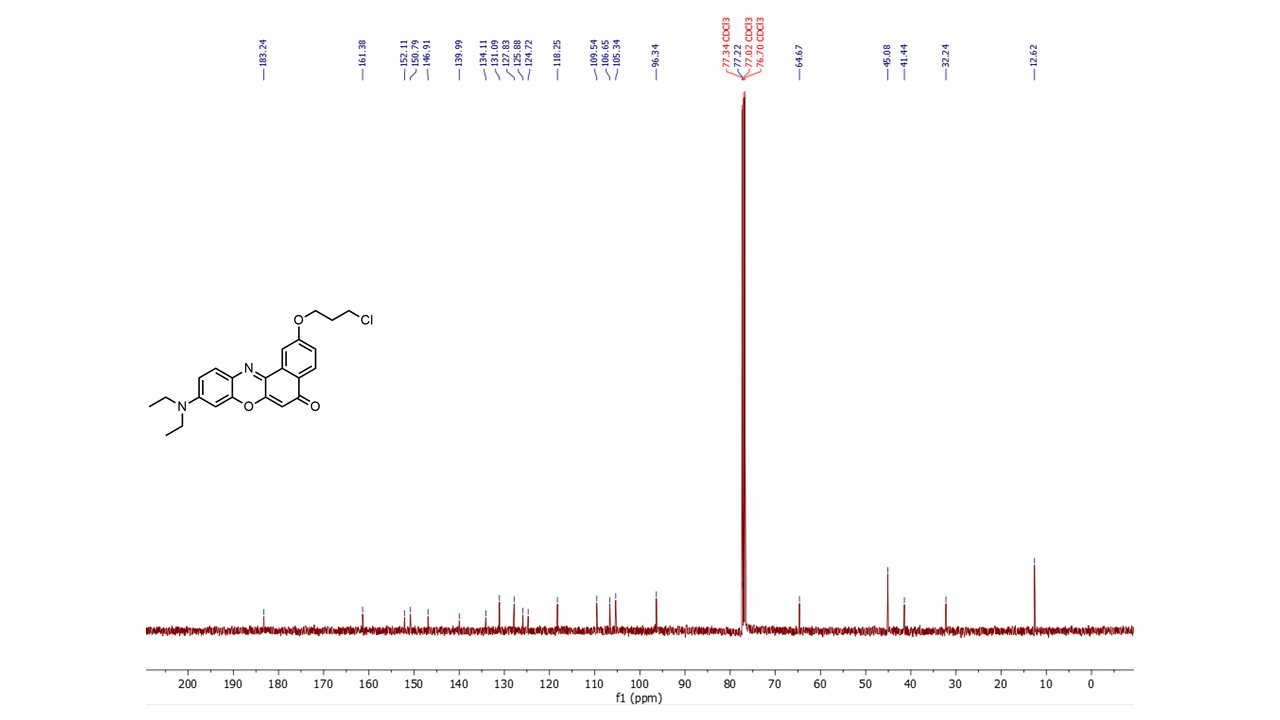


**Fig. S13 ^13^C NMR spectrum of 2-(3-Chloropropoxy)-9-(diethylamino)-5H-benzo[a]phenoxazin-5-one (6)**


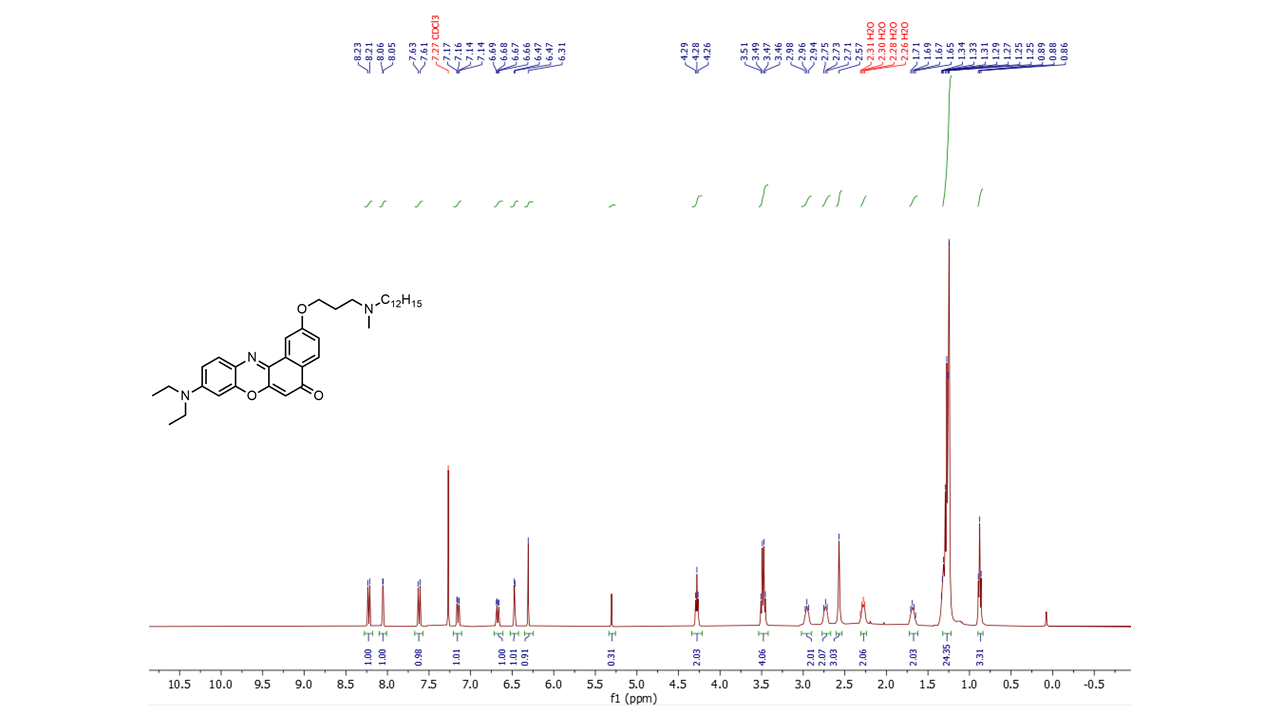


**Fig. S14 ^1^H NMR spectrum of 9-(Diethylamino)-2-[3-(dodecylmethylamino)propoxy]-5H-benzo[a]phenoxazin-5-one (7)**


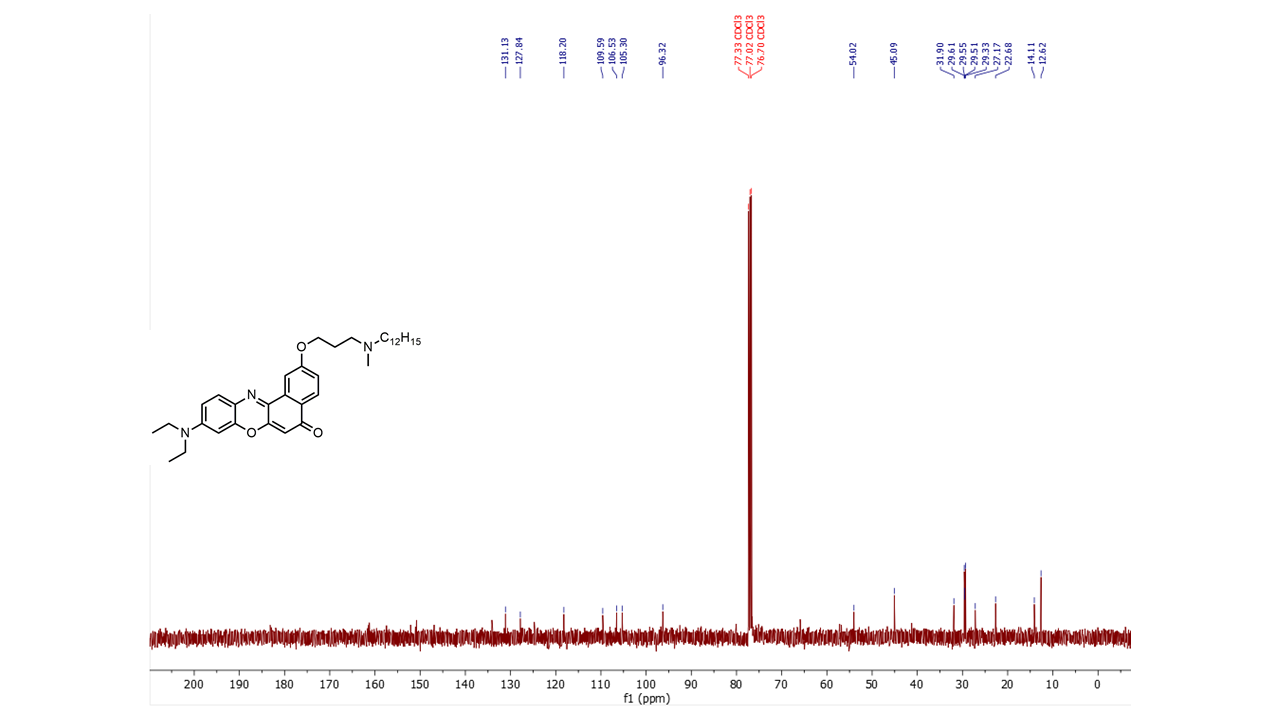


**Fig. S15 ^13^C NMR spectrum of 9-(Diethylamino)-2-[3-(dodecylmethylamino)propoxy]-5H-benzo[a]phenoxazin-5-one (7)**


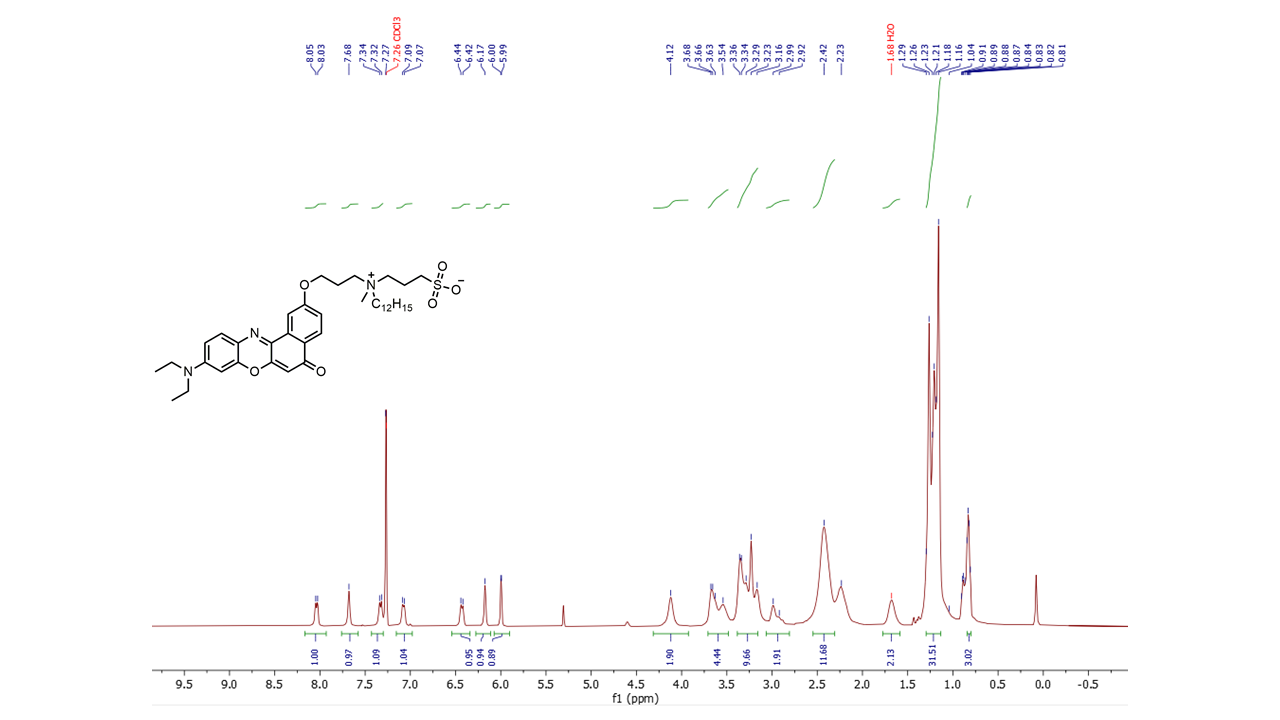


**Fig. S16 ^1^H NMR spectrum of NR12S**


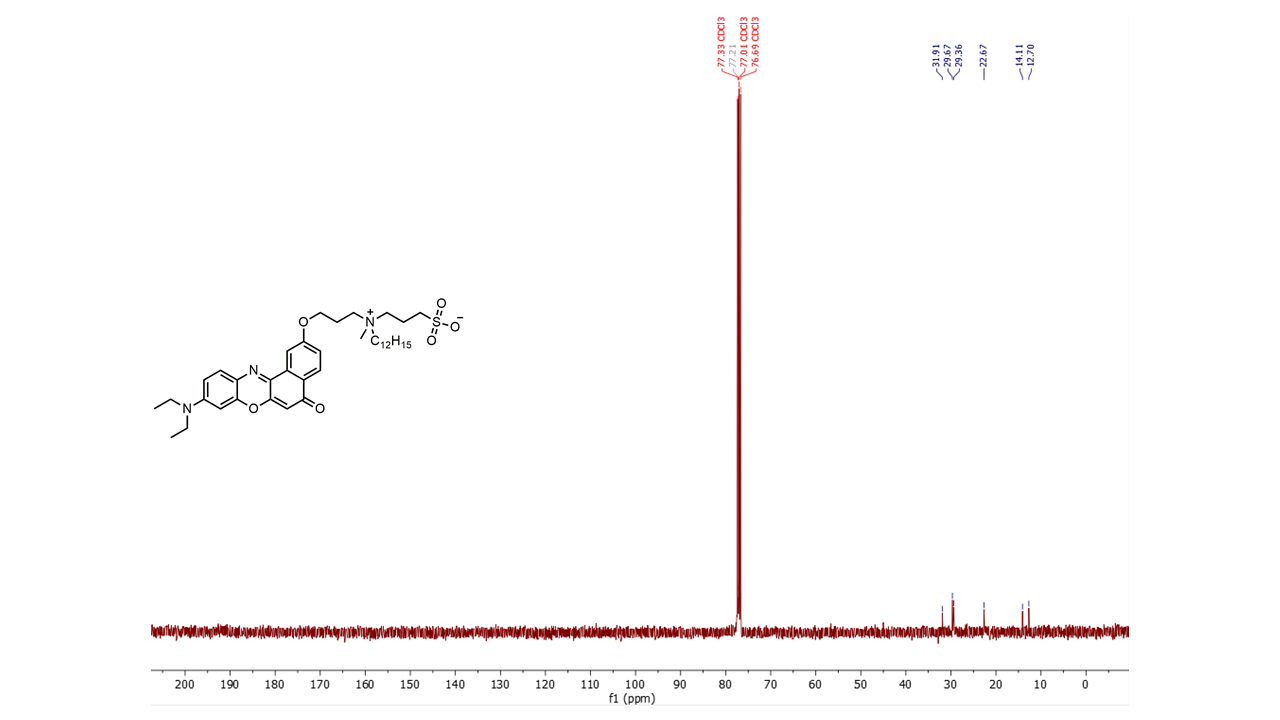


**Fig. S17 ^13^C NMR spectrum of NR12S**


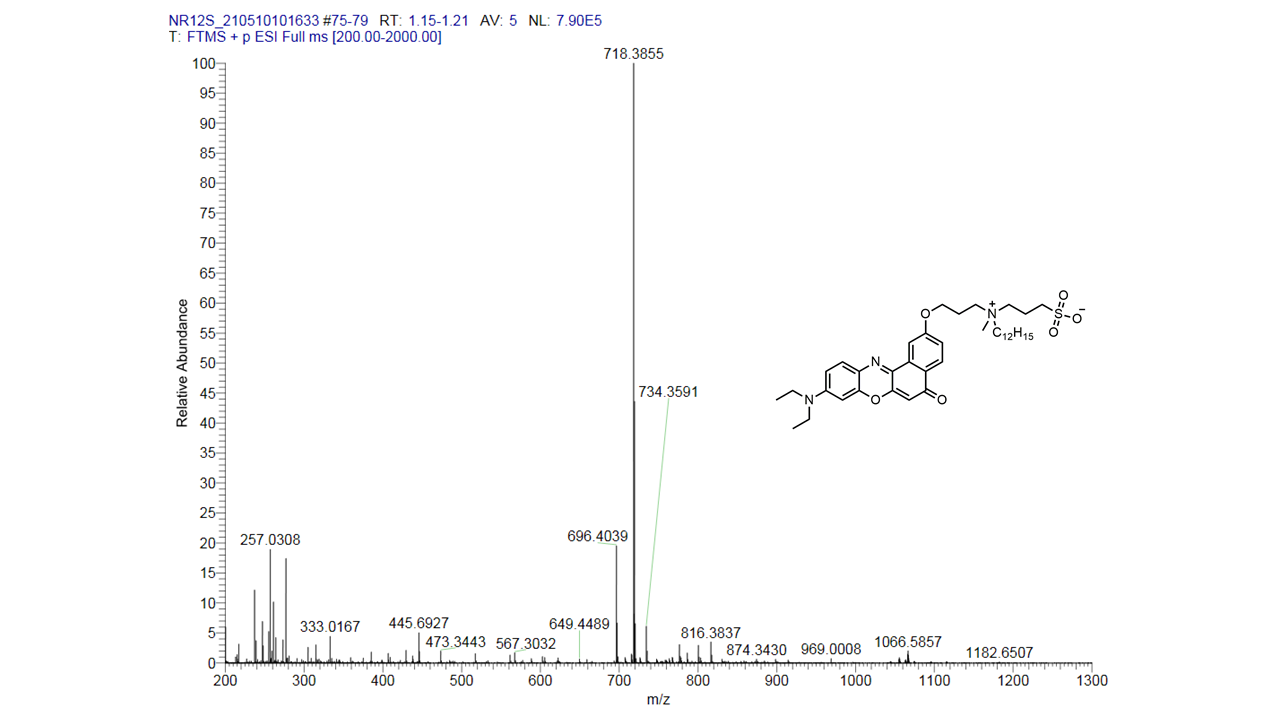


**Fig. S18 HRMS (ESI) of NR12S**


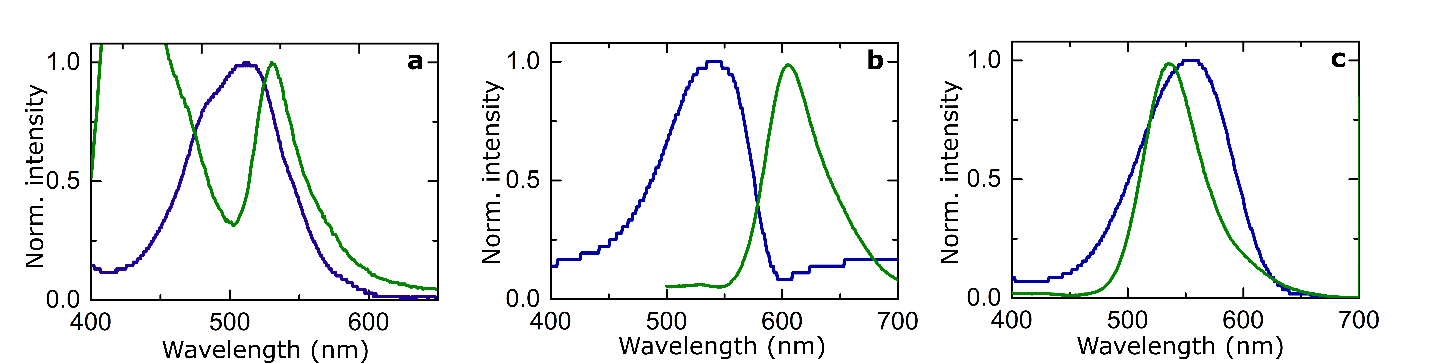


**Fig. S19 Spectroscopic characterization of CWP-BDP and NR12S.** Absorbance (blue) and fluorescence emission (green) spectra of (a) CWP-BDP in chloroform, (b) NR12S in chloroform, (c) NR12S in methanol.

**Methods S1 Synthesis protocol of CWP-BDP**

All chemicals were purchased from Sigma-Aldrich, Fischer Scientific or TCI Chemicals and used as received unless indicated otherwise. 2-Methylpyrrole was purchased from Oxchem Corporation. All oxygen-sensitive reactions were performed under nitrogen atmosphere and slightly negative pressure. Column chromatography was performed on silica gel of 40–63 μm particle size. Where the reaction solvent or eluent consisted of mixture of solvents, the ratios are reported by volume. ^1^H and ^13^C spectra were recorded on a Bruker Avance III 400MHz spectrometer. Mass spectra were recorded using ElectroSpray Ionization (ESI) on a Thermo Scientific Exactive mass spectrometer.

*10-(4-(bromomethyl)phenyl)-5,5-difluoro-3,7-dimethyl-5H-dipyrrolo [1,2-c:2',1'-f] [1,3,2] diazaborinin-4-ium-5-uide (1)*

Freshly distilled 2-methylpyrrole (421 μL, 5 mmol) and 4-(bromomethyl)benzaldehyde (500 mg, 2.5 mmol) were added to anhydrous dichloromethane (130 mL) and the mixture was degassed by sparging with nitrogen for 30 min before addition of TFA (100 μL, 1.3 mmol). The mixture was stirred for 2h at room temperature. 2,3-dichloro-5,6-dicyano-1,4-benzoquinone (570 mg, 2.5 mmol) was added and the mixture degassed by sparging with nitrogen for 10 min. The mixture was stirred further for 20 min. Diisopropylethylamine (3.06 mL, 17.6 mmol) was added, followed by the addition of boron trifluoride diethyl etherate (3.1 mL, 25.1 mmol). The reaction was stirred at room temperature for 24h. The solvent was evaporated at 40°C under reduced pressure to give a dark purple residue that was purified by column chromatography on silica gel (dichloromethane) to give **1** as an orange solid (130 mg, 13% yield). **^1^H NMR** (400 MHz, CDCl_3_) δ 7.48 (m, 4H), 6.71 (d, 2H), 6.28 (d, 2H), 4.56 (s, 2H), 2.65 (s, 6H). **^13^C NMR** (101 MHz, CDCl_3_) δ 157.85, 141.61, 139.67, 134.18, 130.80, 130.30, 128.86, 119.56, 32.48, 14.94. **HRMS** (ESI) calcd for C_18_H_16_BBrF_2_N_2_ [M-H]^-^, 387.0527; found, 387.0486.

*10-(4-(azidomethyl)phenyl)-5,5-difluoro-3,7-dimethyl-5H-dipyrrolo [1,2-c:2',1'-f] [1,3,2] diazaborinin-4-ium-5-uide (2)*

Sodium azide (80 mg, 1.2 mmol) was dissolved in Milli Q water (200 μL) and the solution was added dropwise to anhydrous DMF (5 mL). **1** (100 mg, 0.26 mmol) was added and the mixture was degassed by sparging with nirogen for 15 min. The reaction was stirred at room temperature overnight, cooled down, and diluted with dichloromethane. The organic solution was washed five times with Milli Q water, dried over magnesium sulfate, filtered, and the solvent was removed at 40°C under reduced pressure. The residue was purified by column chromatography on silica gel (petroleum ether:dichloromethane=1:1) to yield **2** as an orange solid (64 mg, 71% yield). **^1^H NMR** (400 MHz, CDCl_3_) δ 7.51 (m, 2H), 7.44 (m, 2H), 6.69 (d, 2H), 6.28 (d, 2H), 4.46 (s, 2H), 2.65 (s, 6H). **^13^C NMR** (101 MHz, CDCl_3_) δ 157.84, 141.71, 137.46, 134.44, 134.08, 130.82, 130.29, 127.83, 119.55, 99.98, 54.37, 14.93. **HRMS** (ESI) calcd for C_18_H_16_BF_2_N_5_ [M-H]^-^, 350.1427; found, 350.1395.

*CWP-BDP (3)*

The cell wall binding peptide (SVHHYKYK) derivative was prepared using standard Fmoc/*t*Bu-based protocols on a Rink amid resin by means of an automated peptide synthesizer. Specifically, the sequence was extended on the N-terminus with four G residues and 4-pentynoic acid in order to separate the cell wall binding peptide from the BODIPY rotor. After acidic cleavage and side-chain deprotection of the resin-bound peptide, the crude alkyne-peptide derivative was subjected to copper-catalysed alkyne-azide cycloaddition (CuAAC) conjugation to the azide-functionalized BODIPY rotor. For this, **2** (4.2 mg; 12 μmol) was mixed with a calculated excess of the expected amount of peptide obtained from the resin (*i.e.* 24.6 mg, 18 μmol) in DMF:water=1:1 (2 mL). CuSO_4_ (20 mg, 90 μmol) and sodium ascorbate (35 mg, 180 μmol) were added. The reaction was performed overnight at room temperature. The crude mixture was subjected to preparative RP-HPLC using a prep-LC-MS system (Alltima, C18, 5μ, 250mm × 22mm; gradient: 0–5 min @A, in 5–25 min to B, 25–30 min @B, in 30–35 min to A, 35–40 min @A [buffer A: 95% MilliQ, 5% MeCN, 0.1% TFA; buffer B: 95% MeCN, 5% MilliQ, 0.1% TFA], *t*_R_ = 15.46 min). The purified fraction was freeze-dried to get **CWP-BDP** (3.3mg, 16% yield). **HRMS** (ESI) calcd for C_81_H_105_BF_2_N_24_O_16_ [M+3H]^3+^, 573.9473; found, 573.9475 and [M+2H]^2+^, 860.4174; found, 860.4167.

**Methods S2 Synthesis protocol of NR12S**

*5-(Diethylamino)-2-nitrosophenol (4)*

M-diethylaminophenol (6 g, 36 mmol) was grinded in a mortar and dissolved in 37% HCl (40 ml). While stirring, the mixture was cooled to 0°C using an ice bath. To the cold solution, a solution of sodium nitrite (2.5 g in 20 ml MQ water) was added dropwise over 40 min, while keeping the temperature between 0-5°C. Once the addition was complete, the reaction mixture was stirred at 0-5°C for 3h. The crude HCl salt was collected by filtration and dried in a vacuum oven overnight. The product was then dissolved in the minimum amount of ethanol, at 100°C, cooled down to 40°C, and diethyl ether was slowly added until the apparition of a solid precipitate. The mixture was then cooled down to room temperature and stored at -20°C overnight for recrystallization to take place. The recrystallized solid was filtered, and dried overnight at 40°C in a vacuum oven, to yield **4** (1.46 g, 21% yield). The yield was much lower than in the reference paper (reported to be 70%), due to an attempt to wash the recrystallized solid with MQ water, which also dissolves the wanted product. **^1^H NMR** (400 MHz, MeOD) δ 7.73 (d, 1H), 7.22 (d, 1H), 6.41 (s, 1H), 3.99 (m, 4H), 1.48 (t, 6H). **^13^C NMR** (101 MHz, MeOD) δ 165.82, 162.64, 144.48, 123.41, 119.59, 13.24, 11.71.

*9-(Diethylamino)-2-hydroxy-5H-benzo[a]phenoxazin-5-one (5)*

**4** (1 g, 5 mmol) and 1,6-dihydroxynaphtalene (0.85 g, 5 mmol) were refluxed (150°C) for 4h in anhydrous DMF (15 ml). This step should have been done under nitrogen atmosphere, which was not specified in the reference paper. The solvent was evaporated under nitrogen flow, and the crude product purified by Silica column chromatography with ethyl acetate:2-propanol=5:2 as eluent to yield **5** (166 mg, 9.6% yield) as a purple solid with green reflections. **^1^H NMR** (400 MHz, DMSO-d6) δ 10.40 (s, 1H), 7.96 (d, 1H), 7.87 (s, 1H), 7.57 (d, 1H), 7.09 (d, 1H), 6.79 (d, 1H), 6.76 (s, 1H), 6.13 (s, 1H), 3.5 (m, 4H), 1.17 (t, 6H). **^13^C NMR** (101 MHz, DMSO-d6) δ 182.83, 161.87, 152.86, 151.94, 147.67, 139.98, 135.02, 132.08, 128.73, 125.12, 119.62, 111.16, 109.38, 105.35, 97.30, 63.29, 45.69, 26.77, 13.77.

*2-(3-Chloropropoxy)-9-(diethylamino)-5H-benzo[a]phenoxazin-5-one (6)*

**5** (110 mg, 0.3 mmol) was dissolved in anhydrous DMF (1.5 ml) and de-oxygenated by bubbling nitrogen for 5 min. The mixture was cooled down to 0-5°C using an ice bath. Potassium carbonate (239 mg, 1.7 mmol) and 1-bromo-3-chloropropane (548 mg, 3 mmol) were added. The reaction flask was sealed and de-oxygenated under nitrogen. The reaction was performed for 4h30 at 70°C. The solvent was then evaporated under nitrogen flow. The resulting crude product was triturated with water, filtered, rinsed once with water, triturated with heptane, and rinsed once with heptane. The filtered compound was dried at 40°C in a vacuum oven overnight to yield **6** (120 mg, 89% yield) as a dark violet solid. **^1^H NMR** (400 MHz, CDCl_3_) δ 8.26 (d, 1H), 8.09 (s, 1H), 7.63 (d, 1H), 7.20 (d, 1H), 6.69 (d, 1H), 6.66 (s, 1H), 6.31 (s, 1H), 4.37 (t, 2H), 3.83 (t, 2H), 3.51 (m, 4H), 2.37 (m, 2H), 1.31 (t, 6H). **^13^C NMR** (101 MHz, CDCl_3_) δ 183.24, 161.38, 152.11, 150.79, 146.91, 139.99, 134.11, 131.09, 127.83, 125.88, 124.72, 118.25, 109.54, 106.65, 105.34, 96.34, 64.67, 45.08, 41.44, 32.24, 12.62.

*9-(Diethylamino)-2-[3-(dodecylmethylamino)propoxy]-5H-benzo[a]phenoxazin-5-one (7)*

**6** (100 mg, 0.2 mmol) and n-dodecylmethylamine (194 mg, 0.97 mmol) were dissolved in 2-butanone (2 ml). The mixture was de-oxygenated by bubbling nitrogen for 5 min. Potassium carbonate (84 mg, 0.6 mmol) and potassium iodide (43 mg, 0.26 mmol) were added. The mixture was further de-oxygenated by bubbling nitrogen and the flask was sealed and put under nitrogen atmosphere. It might be needed to adjust the volume of 2-butanone, so as to dissolve all the reactants and have a volume suitable for nitrogen bubbling, without drying out the mixture and get a solid deposit along the flask walls. The reaction was performed under reflux at 85°C for 48h. The solvent was evaporated and the crude product purified by silica column chromatography using dichloromethane:methanol=95:5 as eluent, to yield **7** (70 mg, 50%) as a dark violet solid. **^1^H NMR** (400 MHz, CDCl_3_) δ 8.23 (d, 1H), 8.06 (s, 1H), 7.63 (d, 1H), 7.17 (d, 1H), 6.69 (d, 1H), 6.66 (s, 1H), 6.31 (s, 1H), 4.29 (t, 2H), 3.51 (m, 4H), 2.98 (m, 2H), 2.75 (m, 2H), 2.57 (s, 3H), 1.71 (m, 2H), 1.34 (m, 24H), 0.86 (t, 3H). **^13^C NMR** (101 MHz, CDCl_3_) δ 131.13, 127.84, 118.20, 109.59, 106.53, 105.30, 96.32, 54.02, 45.09, 31.90, 29.61, 29.55, 29.51, 29.33, 27.17, 22.68, 14.11, 12.62.

*NR12S (8)*

In a well dried, degassed flask, **7** (20 mg, 34.8 µmol) was dissolved in anhydrous acetonitrile (3 ml). The mixture was de-oxygenated by bubbling nitrogen for 5 min. 1,3-propanesultone (50 µl, 57.3 µmol) was added, and the flask was sealed and put under nitrogen atmosphere. The reaction was performed at 80°C for 48h. The solvent was then evaporated and the crude product purified by silica column chromatography using dichloromethane:methanol=85:15 as eluent, to yield **NR12S** (20 mg, 82% yield) as a dark violet solid. **^1^H NMR** (400 MHz, CDCl_3_) δ 8.05 (d, 1H), 7.68 (s, 1H), 7.34 (d, 1H), 7.09 (d, 1H), 6.44 (d, 1H), 6.17 (s, 1H), 6.00 (s, 1H), 4.12 (s, 2H), 3.68-3.54 (m, 4H), 3.36-3.16 (m, 7H), 2.99-2.92 (m, 2H), 2.42 (m, 6H), 1.29-1.16 (m, 24H), 0.84 (m, 3H). **HRMS** (ESI) calcd for C_39_H_58_N_3_O_6_S+ [M+H]^+^: 696.40; found, 696.4039.


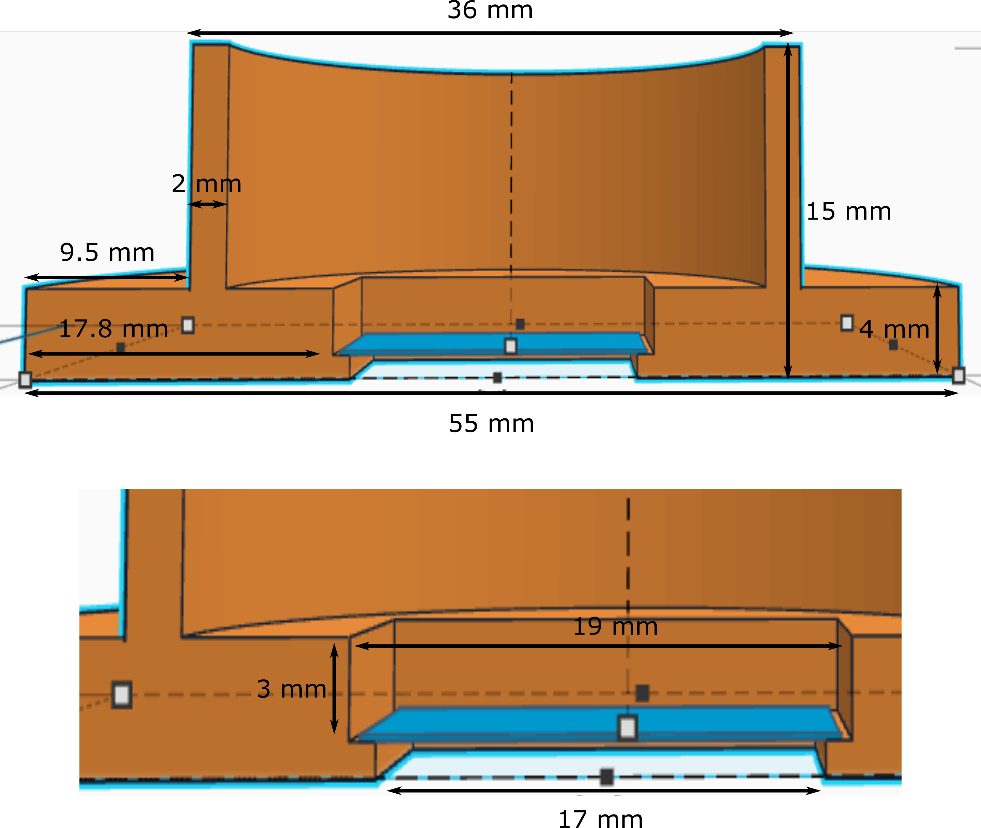


**Fig. S20 Schematic of the 3D-printed observation chambers used for all cell imaging experiments.** The .stl files for 3D-printing are available for download at:

https://github.com/jorissprakel/Phytophthora_chambers

**Table S1** **Experimental repeats and number of specimen imaged**

| Experiments | Figures | Sample numbers |
| --- | --- | --- |
| Growth of germinated cysts | Fig. **1d,e**  Fig. **2d**  Fig. **4a,e**  Fig. **5a,d**  Fig. **S25**-**S27** | Non-invasive: N=30 cells from 3 cell batches for each probe; invasive: N=30 cells from 3 cell batches for CWP-BDP |
| DMSO | Fig. **S31** | N=30 cells from 2 cell batches for each probe |
| Giant synthetic vesicles | Fig. **2h**  Fig. **4i,j**  Fig. **S30** | DOPC:SM=1:1 (molar ratio): N=20 vesicles from 3 vesicle batches for NR12S; DOPC:SM:cholesterol=1:1:0.7 (molar ratio): N=20 vesicles from 3 vesicle batches for NR12S; vesicles supplemented with fluopicolide: N=20 vesicles from 2 vesicle batches for each vesicle type (cholesterol-free and cholesterol-rich), for NR12S |
| Mycelium | Fig. **2f** | Supplemented with 0.2 µg ml^-1^ β-sitosterol: N=3 mycelia for NR12S |
| Hypo-osmotic shock | Fig. **3a,b,d,e**  Fig. **S28** | N=20 cells from 2 cell batches for each probe |
| Fluopicolide | Fig. **4b,f**  Fig. **S29** | N=30 cells from 3 cell batches |
| Valifenalate | Fig. **4c,g**  Fig. **S32** | N=30 cells from 3 cell batches for each probe |
| Latrunculin B | Fig. **5b,e**  Fig. **S33** | N=30 cells from 2 cell batches for each probe |
| Oryzalin | Fig. **5c,f**  Fig. **S34** | N=30 cells from 2 cell batches for each probe |
| Autofluorescence control | Fig. **S21** | 2 cell batches for both single-photon and two-photon excitation set-ups |
| Growth rate assay | Fig. **S24** | 1 cell batch for each condition, N=96 cells for control, N=103 cells for NR12S, N=25 cells for CWP-BDP |
| Invasivity assays | n.a. | 1 cell batch for each condition, N=138 cells for control, N=84 cells for fluopicolide, N=103 cells for valifenalate, N=3 cells for LatB, N=201 cells for oryzalin |


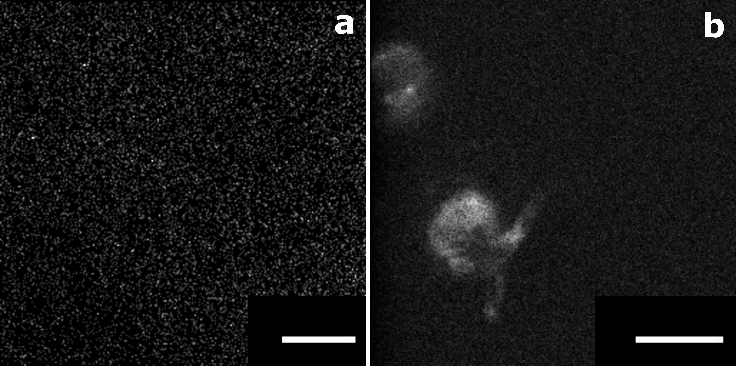


**Fig. S21 Autofluorescence signal of the** ***Phytophthora infestans* germlings.** (a) Autofluorescence obtained by single-photon excition on the TCS SP8, with a laser intensity ten times higher than the maximum laser intensity used for CWP-BDP excitation. Only noise was imaged. Scale bar = 50 µm. (b) Autofluorescence obtained by two-photon excitation on the TCS SP8 multiphoton, with the maximum laser intensity used for CWP-BDP and NR12S excitation. The autofluorescence signal is low and only visible inside the cell, not within the cell wall and plasma membrane. Scale bar = 10 µm.


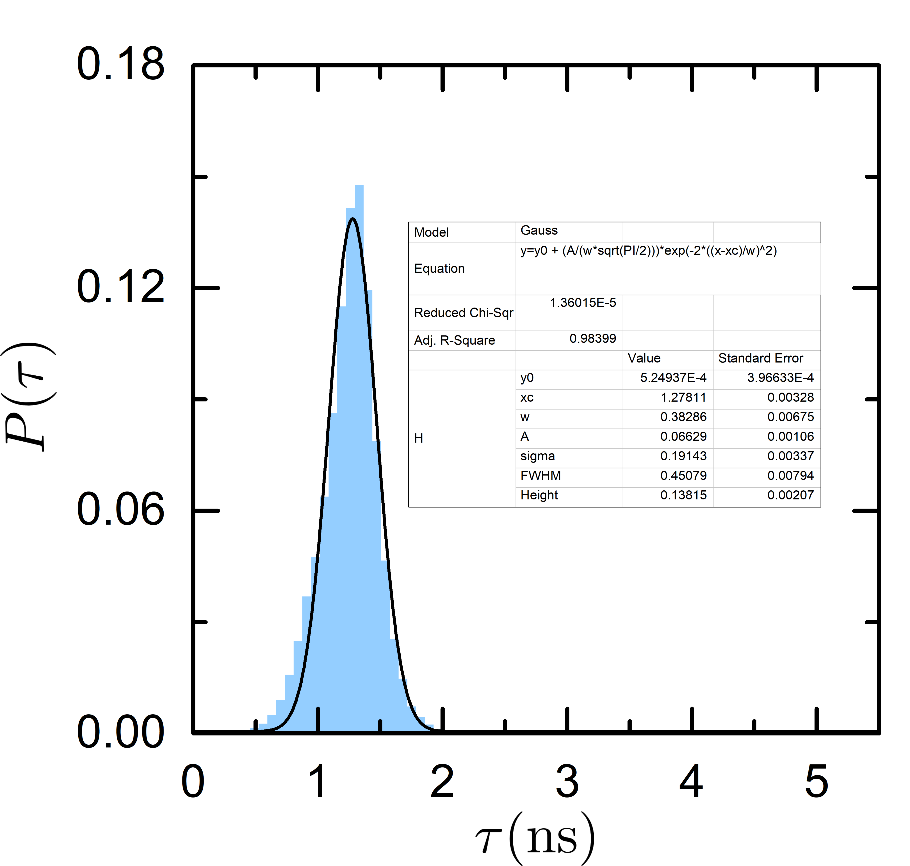


**Fig. S22 Fluorescence lifetime distribution of CWP-BDP in water.** The fluorescence lifetime distribution obtained in a homogeneous aqueous solution is fitted with a Gaussian to determine the noise threshold of the probe.


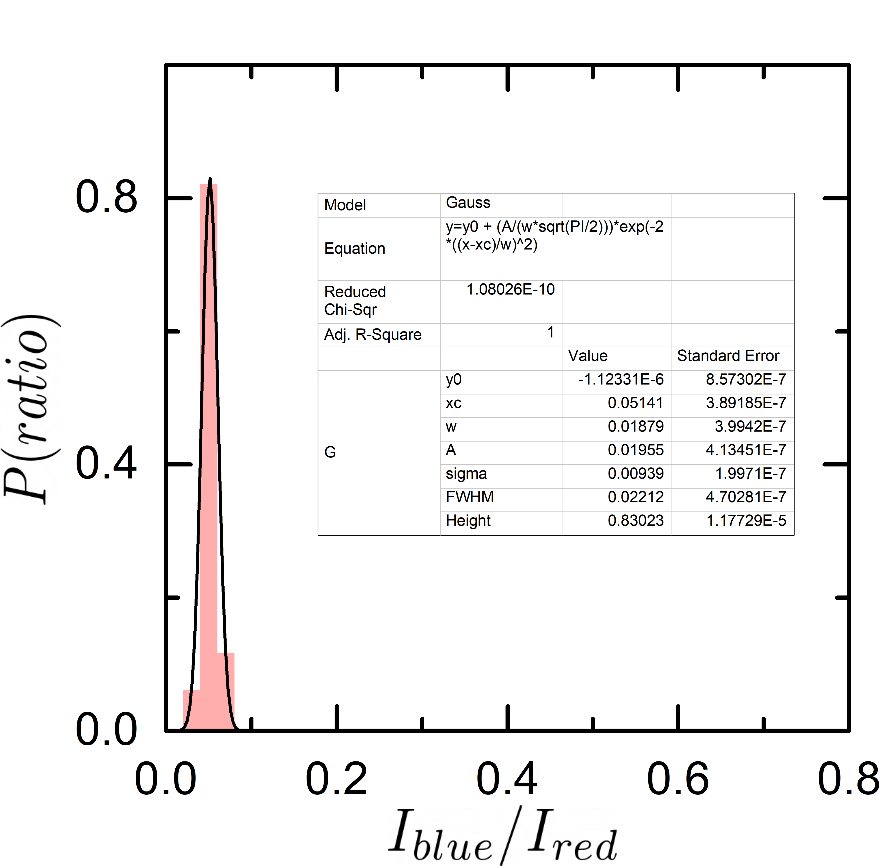


**Fig. S23 Intensity ratio distribution of NR12S in chloroform.** The intensity ratio distribution obtained in a homogeneous solution in chloroform is fitted with a Gaussian to determine the noise threshold of the probe. The same experiment performed in methanol or dimethylsulfoxide does not allow to plot a ratio distribution given the very low photon count obtained in the blue channel.


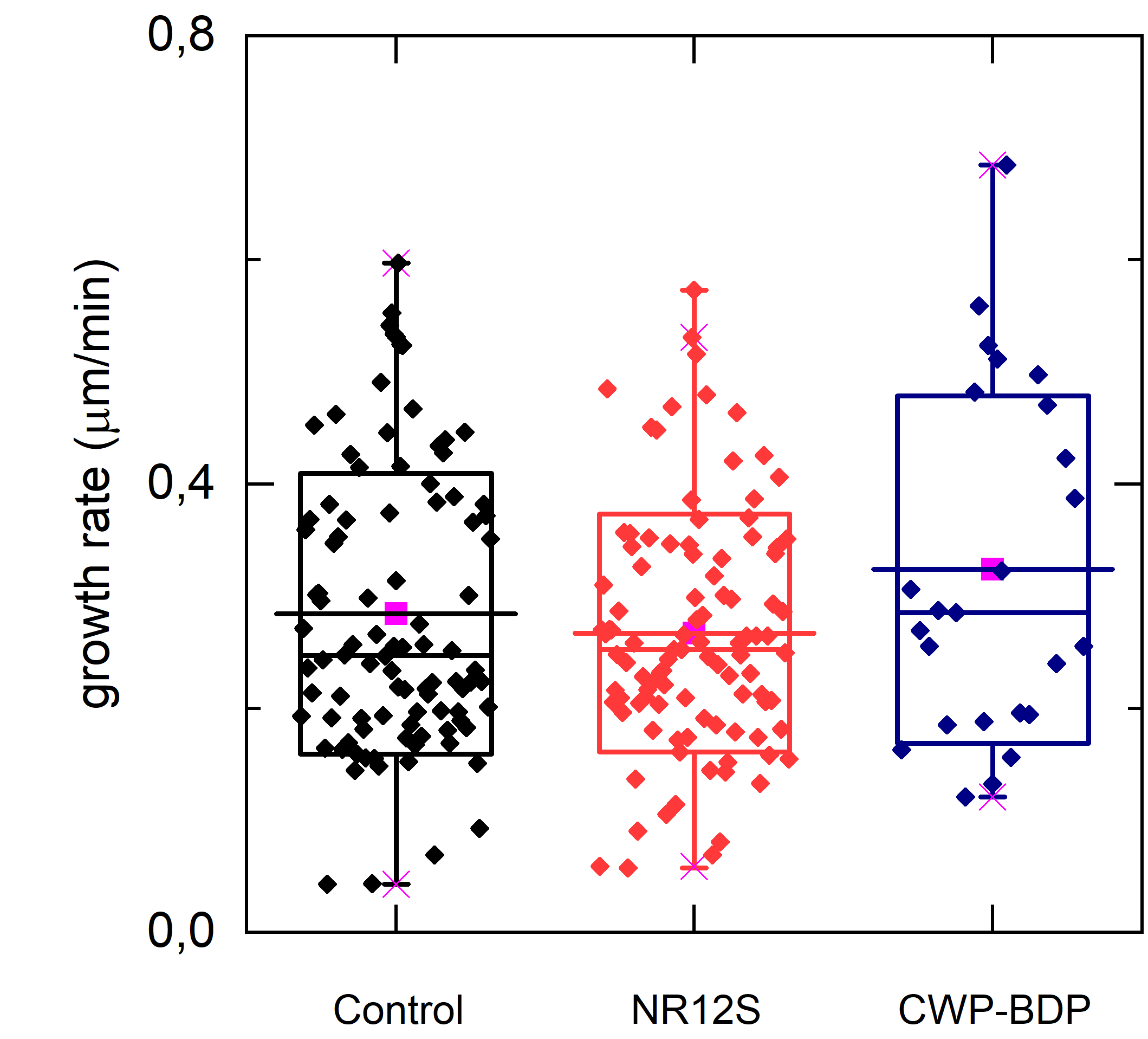


**Fig. S24 Box plot of the germ tube growth rate in absence or presence of NR12S and CWP-BDP respectively.** The presence of 10 µmol l^-1^ NR12S or CWP-BDP does not affect the growth rate of germ tubes. Untreated cells have a extension rate of 0.28 ± 0.12 (std deviation) µm/min, NR12S treated cells have an extension rate of 0.27 ± 0.04 µm/min, and CWP-BDP treated cells an extension rate of 0.32 ± 0.17 µm/min. The calculated differences fall within error.


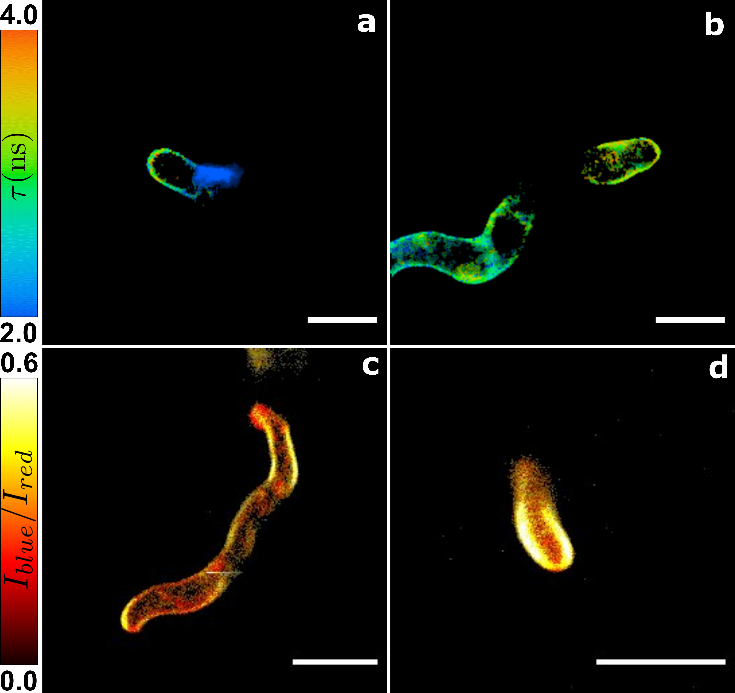


**Fig. S25 Mapping of spatial variations in the cell wall mesh size and plasma membrane chemical polarity of *Phytophthora* *infestans* germlings.** (a, b) Fluorescence lifetime mesh size map of control germlings growing in water 1 hour post application. (c, d) Intensity ratio chemical polarity map of control germlings growing in water 1 hour post application. Scale bars = 10 µm.


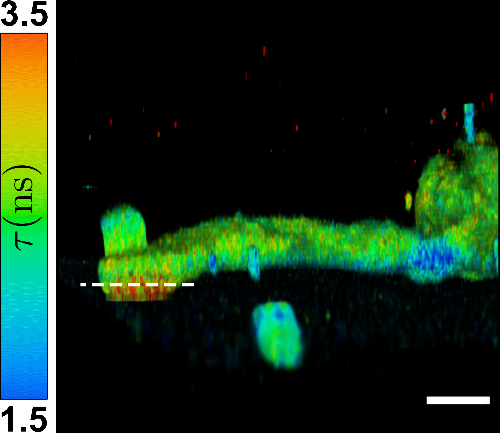


**Fig. S26 3D-mapping of spatial variations in the cell wall mesh size of *Phytophthora* *infestans* germlings.** 3D fluorescence lifetime mesh size map of *Phytophthora* *infestans* germlings upon interaction with the host surface. Scale bar = 5 µm.


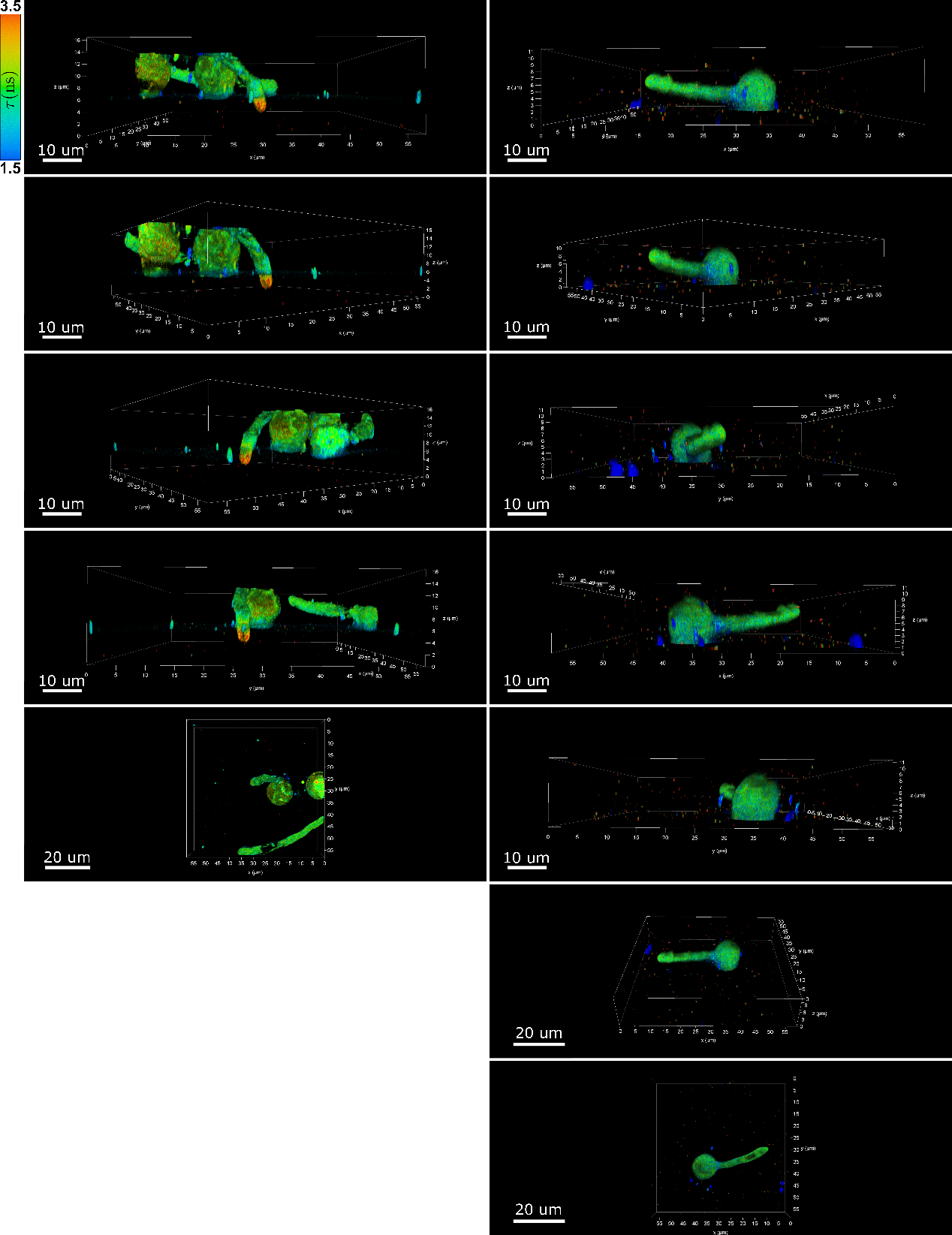


**Fig. S27 3D-mapping of spatial variations in the cell wall mesh size of *Phytophthora* *infestans* germlings.** Rotation views of the 3D fluorescence lifetime mesh size maps of *Phytophthora* *infestans* germlings represented in Fig. **1**.


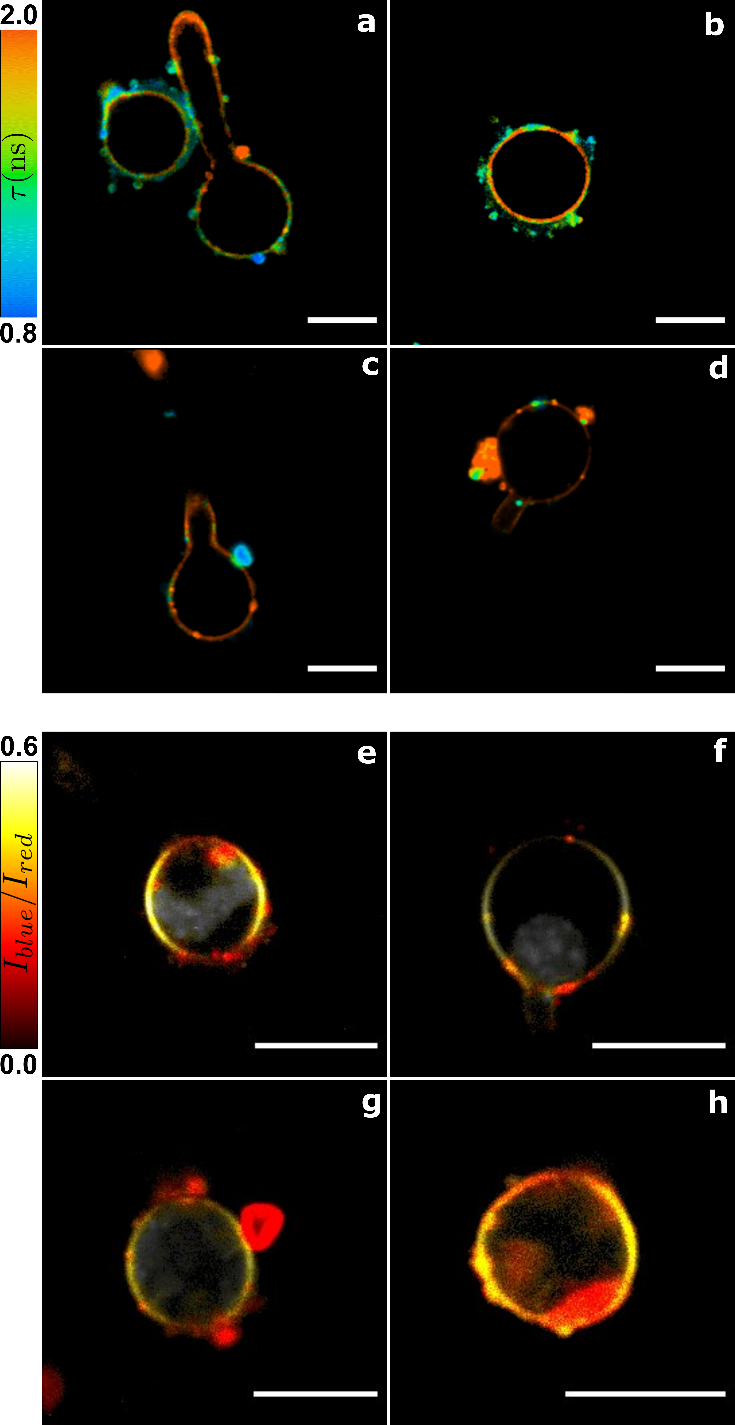


**Fig. S28 Hypo-osmotic treatment on *Phytophthora* *infestans* germlings, and effect on their cell wall and plasma membrane mechano-chemical properties.** (a, b) Fluorescence lifetime mesh size map of germlings growing in a 90 mmol l^-1^ PEG2000g mol^-1^ aqueous solution. (c, d) Fluorescence lifetime mesh size map of germlings after transfer from the 90 mmol l^-1^ PEG2000g mol^-1^ aqueous solution to water. (e, f) Intensity ratio chemical polarity map of germlings growing in a 90 mmol l^-1^ PEG2000g mol^-1^ aqueous solution. (g, h) Intensity ratio chemical polarity map of germlings after transfer from the 90 mmol l^-1^ PEG2000g mol^-1^ aqueous solution to water. Scale bars = 10 µm.


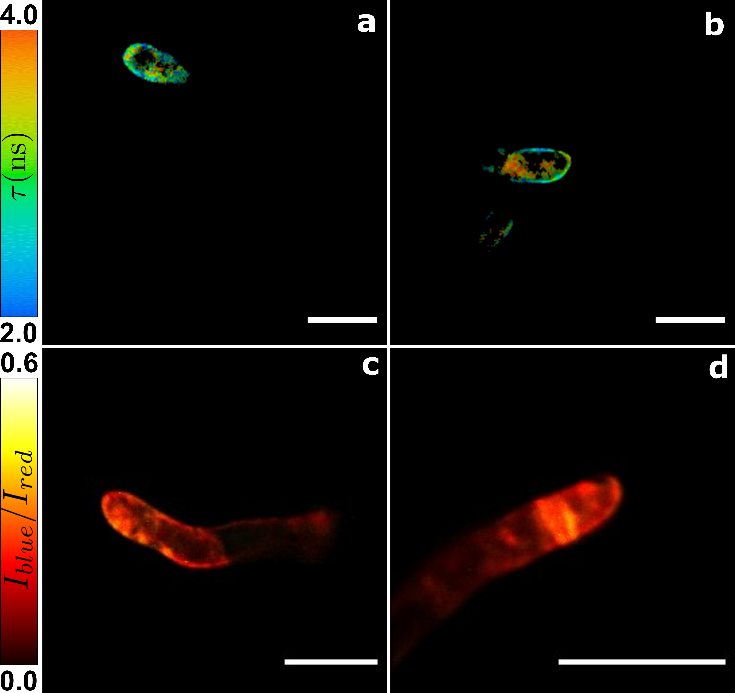


**Fig. S29 Mapping of spatial variations induced by treatment with 25 nmol l^-1^ fluopicolide in the cell wall mesh size and plasma membrane chemical polarity of *Phytophthora* *infestans* germlings.** (a, b) Fluorescence lifetime mesh size map of germlings growing in water 1 hour post application, and treated with 25 nmol l^-1^ fluopicolide for 1h. (c, d) Intensity ratio chemical polarity map of germlings growing in water 1 hour post application, and treated with 25 nmol l^-1^ fluopicolide for 1h. Scale bars = 10 µm.


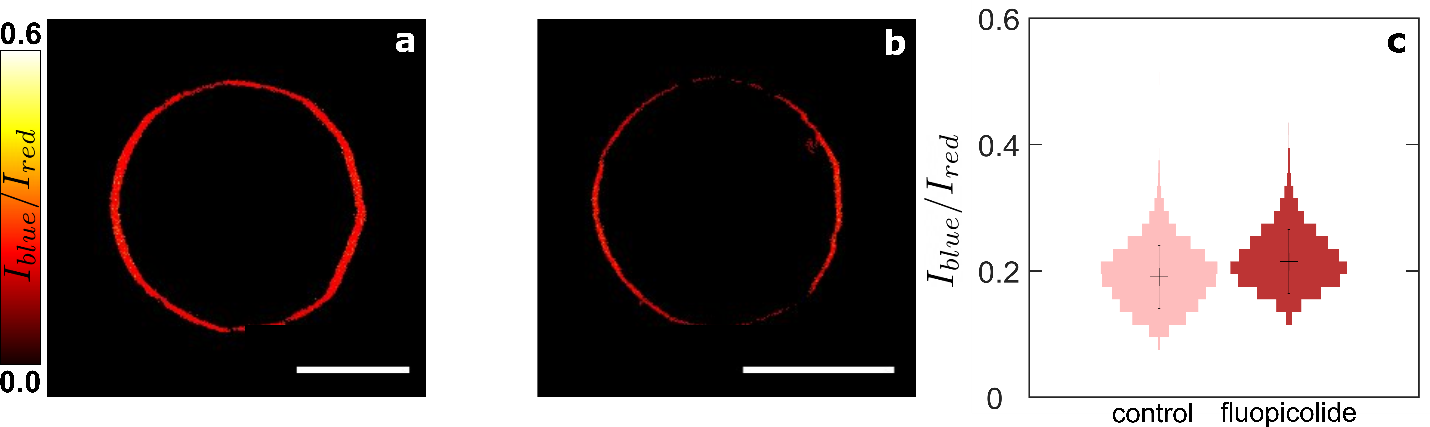


**Fig. S30 Mapping of spatial variations in plasma membrane chemical polarity in DOPC:SM synthetic vesicles.** (a) Intensity ratio chemical polarity map of 1,2-dioleoyl-sn-glycero-3-phosphocholine (DOPC):sphingomyelin (SM) = 1:1 (molar ratio) synthetic vesicles. (b) Intensity ratio chemical polarity map of the same vesicles incubated with 25 nmol l^-1^ fluopicolide. (c) Corresponding intensity ratio probability distributions. Scale bars = 10 µm.


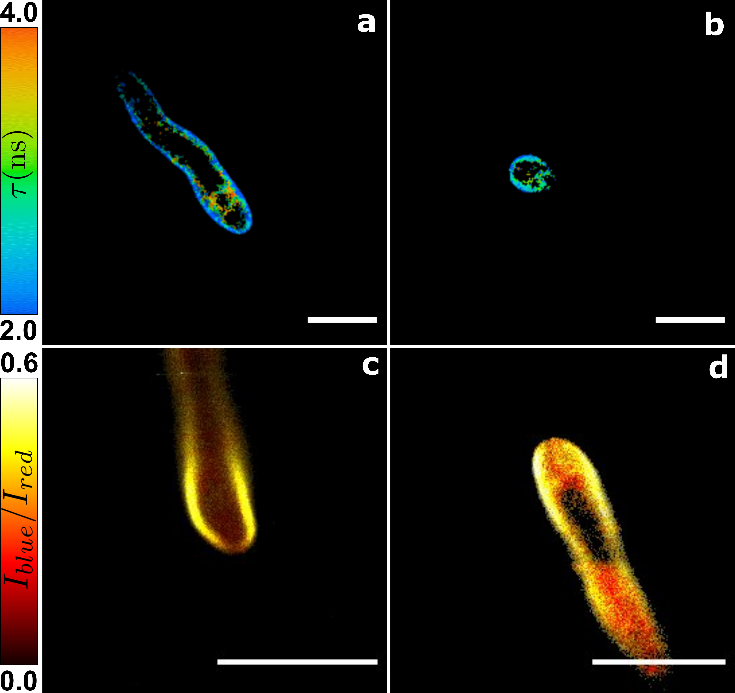


**Fig. S31 Mapping of spatial variations induced by treatment with 1% (v/v) DMSO in the cell wall mesh size and plasma membrane chemical polarity of *Phytophthora* *infestans* germlings.** (a, b) Fluorescence lifetime mesh size map of germlings growing in water 1 hour post application, and treated with 1% (v/v) DMSO for 1h. (c, d) Intensity ratio chemical polarity map of germlings growing in water 1 hour post application, and treated with 1% (v/v) DMSO for 1h. Scale bars = 10 µm.


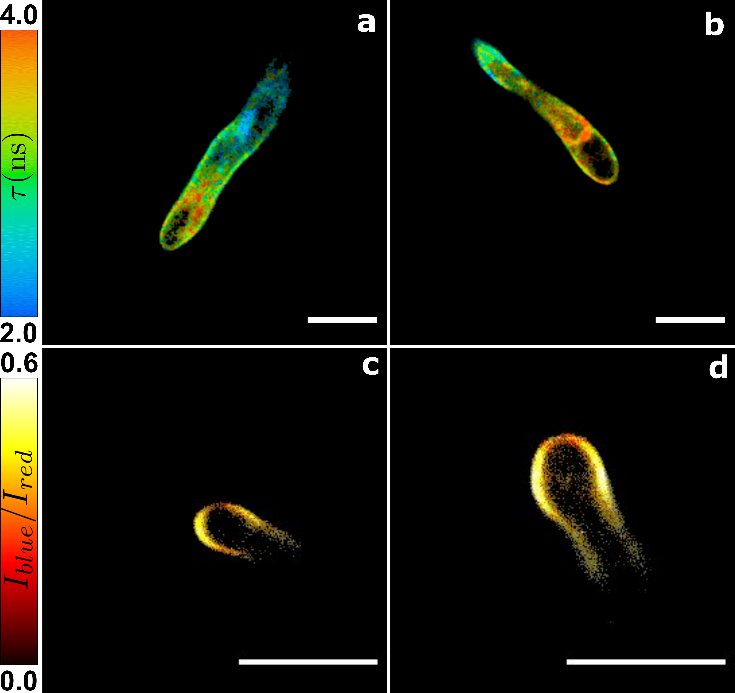


**Fig. S32 Mapping of spatial variations induced by treatment with 125 nmol l^-1^ valifenalate in the cell wall mesh size and plasma membrane chemical polarity of *Phytophthora* *infestans* germlings.** (a, b) Fluorescence lifetime mesh size map of germlings growing in water 1 hour post application, and treated with 125 nmol l^-1^ valifenalate for 1h. (c, d) Intensity ratio chemical polarity map of germlings growing in water 1 hour post application, and treated with 125 nmol l^-1^ valifenalate for 1h. Scale bars = 10 µm.


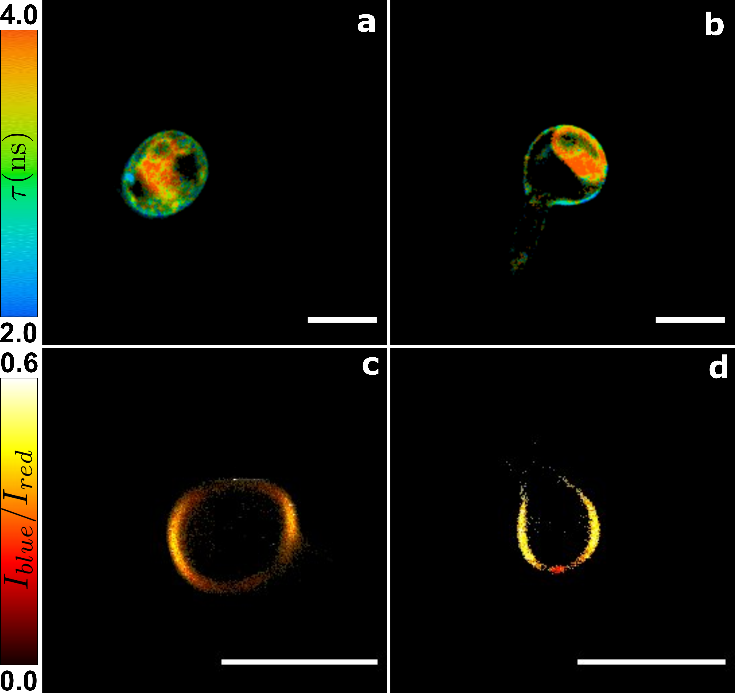


**Fig. S33 Mapping of spatial variations induced by treatment with 1 µmol l^-1^ latrunculin B in the cell wall mesh size and plasma membrane chemical polarity of *Phytophthora infestans* germlings.** (a, b) Fluorescence lifetime mesh size map of germlings growing in water 1 hour post application, and treated with 1 µmol l^-1^ latrunculin B for 1h. (c, d) Intensity ratio chemical polarity map of germlings growing in water 1 hour post application, and treated with 1 µmol l^-1^ latrunculin B for 1h. Scale bars = 10 µm.


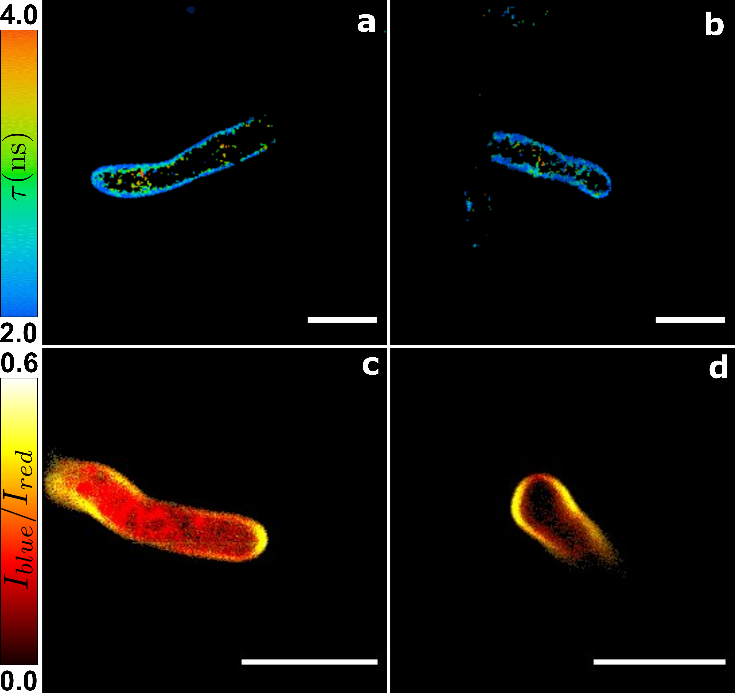


**Fig. S34 Mapping of spatial variations induced by treatment with 0.1 µmol l^-1^ oryzalin in the cell wall mesh size and plasma membrane chemical polarity of *Phytophthora infestans* germlings.** (a, b) Fluorescence lifetime mesh size map of germlings growing in water 1 hour post application, and treated with 0.1 µmol l^-1^ oryzalin for 1h. (c, d) Intensity ratio chemical polarity map of germlings growing in water 1 hour post application, and treated with 0.1 µmol l^-1^ oryzalin for 1h. Scale bars = 10 µm.
